# Comprehensive Analysis of the Immunogenomics of Triple-Negative Breast Cancer Brain Metastases From LCCC1419

**DOI:** 10.3389/fonc.2022.818693

**Published:** 2022-07-27

**Authors:** Eric D. Routh, Amanda E. D. Van Swearingen, Maria J. Sambade, Steven Vensko, Marni B. McClure, Mark G. Woodcock, Shengjie Chai, Luz A. Cuaboy, Amy Wheless, Amy Garrett, Lisa A. Carey, Alan P. Hoyle, Joel S. Parker, Benjamin G. Vincent, Carey K. Anders

**Affiliations:** ^1^ Lineberger Comprehensive Cancer Center, University of North Carolina at Chapel Hill, Chapel Hill, NC, United States; ^2^ National Cancer Center Research Institute, Tokyo, Japan; ^3^ Department of Medicine, Division of Medical Oncology, University of North Carolina at Chapel Hill, Chapel Hill, NC, United States; ^4^ Curriculum in Bioinformatics and Computational Biology, UNC School of Medicine, Chapel Hill, NC, United States; ^5^ Department of Genetics, University of North Carolina at Chapel Hill, Chapel Hill, NC, United States; ^6^ Department of Microbiology and Immunology, University of North Carolina at Chapel Hill, Chapel Hill, NC, United States; ^7^ Division of Hematology, University of North Carolina at Chapel Hill, Chapel Hill, NC, United States

**Keywords:** triple-negative breast cancer, brain metastases, immunogenomics, whole-exome sequencing, mRNA sequencing, biobank

## Abstract

**Background:**

Triple negative breast cancer (TNBC) is an aggressive variant of breast cancer that lacks the expression of estrogen and progesterone receptors (ER and PR) and HER2. Nearly 50% of patients with advanced TNBC will develop brain metastases (BrM), commonly with progressive extracranial disease. Immunotherapy has shown promise in the treatment of advanced TNBC; however, the immune contexture of BrM remains largely unknown. We conducted a comprehensive analysis of TNBC BrM and matched primary tumors to characterize the genomic and immune landscape of TNBC BrM to inform the development of immunotherapy strategies in this aggressive disease.

**Methods:**

Whole-exome sequencing (WES) and RNA sequencing were conducted on formalin-fixed, paraffin-embedded samples of BrM and primary tumors of patients with clinical TNBC (*n* = 25, *n* = 9 matched pairs) from the LCCC1419 biobank at UNC—Chapel Hill. Matched blood was analyzed by DNA sequencing as a comparison for tumor WES for the identification of somatic variants. A comprehensive genomics assessment, including mutational and copy number alteration analyses, neoantigen prediction, and transcriptomic analysis of the tumor immune microenvironment were performed.

**Results:**

Primary and BrM tissues were confirmed as TNBC (23/25 primaries, 16/17 BrM) by immunohistochemistry and of the basal intrinsic subtype (13/15 primaries and 16/19 BrM) by PAM50. Compared to primary tumors, BrM demonstrated a higher tumor mutational burden. *TP53* was the most frequently mutated gene and was altered in 50% of the samples. Neoantigen prediction showed elevated cancer testis antigen- and endogenous retrovirus-derived MHC class I-binding peptides in both primary tumors and BrM and predicted that single-nucleotide variant (SNV)-derived peptides were significantly higher in BrM. BrM demonstrated a reduced immune gene signature expression, although a signature associated with fibroblast-associated wound healing was elevated in BrM. Metrics of T and B cell receptor diversity were also reduced in BrM.

**Conclusions:**

BrM harbored higher mutational burden and SNV-derived neoantigen expression along with reduced immune gene signature expression relative to primary TNBC. Immune signatures correlated with improved survival, including T cell signatures. Further research will expand these findings to other breast cancer subtypes in the same biobank. Exploration of immunomodulatory approaches including vaccine applications and immune checkpoint inhibition to enhance anti-tumor immunity in TNBC BrM is warranted.

## Introduction

Triple-negative breast cancer (TNBC) lacks the expression of hormone receptors estrogen (ER) and progesterone (PR) as well as human epidermal growth factor receptor 2 (HER2). TNBC is also the most aggressive subtype of breast cancer, with a predilection for brain metastases; up to 50% of patients with metastatic TNBC will develop brain metastases (BrM) during their disease course (1). Patients with TNBC BrM face a poor prognosis with a median survival following diagnosis of less than 6 months (2). Despite progress in the treatment of ER+ and HER2+ breast cancer BrM with the advent of brain-penetrant targeted therapies, the prognosis for TNBC BrM remains largely unchanged over the past decade (3). Thus, studies to better understand the biology of TNBC BrM to identify new therapeutic targets are needed.

Previous studies have conducted sequencing of primary and metastatic tissues, including BrM, from melanoma (4), lung cancer (5), breast cancer (6–8), and multiple solid tumor types (9). A seminal work by Brastianos et al. demonstrated that some solid tumors (including BrM) undergo branched evolution during the metastatic process. These studies have led to a growing appreciation that BrM can be biologically different from not just their primary tumors but also extracranial metastases, including differential acquisition or loss of targetable alterations. Breast cancer brain metastasis (BCBrM) have demonstrated mutations and/or copy number alterations in clinically targetable genes and pathways such as HER2 (6, 7, 9), BRAF (8), PI3K/Akt (9), CDK (6), ATM (8), and CRYAB (10) not seen in primary tumors. BrM can also be metabolically different from primaries, with increased oxidative phosphorylation (4). Preclinical studies have demonstrated that these targets can alter the brain metastatic potential and/or growth of BrM in breast cancer models (4, 11–13). These findings have led to the first genomically guided clinical trial in BrM to match alterations present in BrM to an appropriate brain-penetrant inhibitor (NCT03994796).

While these and other prior studies have made significant progress in the genomic characterization of BrM in recent years, no studies have yet focused exclusively on TNBC BrM specifically, and few studies have looked at comprehensive RNA and DNA sequencing-derived features of the tumor immune microenvironment. Using the LCCC1419 Biobank of metastatic breast cancer samples, we have collected and analyzed BrM, matched primaries, and normal tissue from 25 patients with TNBC through both whole-exome sequencing (WES) and mRNA sequencing. We report the somatic mutational landscape of TNBC BrM compared to primary tumors and implement a comprehensive neoantigen prediction pipeline to elucidate potentially immunogenic peptides arising from traditional and alternative neoantigen sources. Utilizing mRNA gene expression, we evaluated the tumor immune microenvironment of TNBC BrM relative to primary tumors and correlate these features with overall survival. To our knowledge, this study represents the largest evaluation of TNBC BrM through WES and RNA-seq and is the first to analyze gene expression and immunogenomics in addition to the mutational landscape.

## Materials and Methods

### Patient Consent and Tissue Collection

Archival formalin-fixed, paraffin embedded (FFPE) tumor tissues were obtained from patients with clinically determined triple-negative breast cancer (TNBC) based on either a primary or metastatic site, with known metastasis to the brain. The patients consented to participation in either the UNC Health Registry (UNC IRB 09-0605), opened on 04/16/2009 and consented between 11/2014 and 06/2016, or to a clinically annotated biobank study at the University of North Carolina at Chapel Hill under an Institutional Review Board (IRB)-approved protocol (LCCC1419) which opened on 10/31/2014 and consented from 11/2014 to 11/2018. Brain metastases tissues were available from *n* = 19 patients (*n* = 19 with RNA, *n* = 17 with DNA), while primary tumor tissue was available from *n* = 16 patients (*n* = 15 with RNA, *n* = 13 with DNA). Matched whole blood samples were available for *n* = 22 patients (DNA). Of these cases, *n* = 9 included matched RNA primary TNBC and BrM tissue pairs from the same patient, and *n* = 6 had matched DNA triplet samples (primary, BrM, and blood), all *n* = 6 of which also had RNA for both primary and BrM samples.

### DNA Whole-Exome Sequencing and Variant Calling

FFPE tumor/tissue blocks and normal fresh frozen blood samples were collected and inventoried through honest brokers in accordance with IRB standards through the UNC Health Registry. UNC patient samples were inventoried through UNC Surgical Pathology Core (SP), while patient samples from outside UNC were inventoried through UNC Tissue Procurement Facility. Tissue blocks were sectioned by UNC Translational Pathology Laboratory (TPL). Twenty-two sections were made at a time: two 5-μm sections at the beginning and end of sectioning for pathologist review and 20 10-μm sections for DNA/RNA isolation on glass slides. TPL pathologists reviewed the samples, circling areas with more than 50% tumor mass for DNA/RNA isolation. When required, the process was repeated to collect more DNA/RNA. UNC BioSpecimen Processing Facility performed all DNA/RNA isolations. DNA from tumor-enriched cores were extracted using the Maxwell 16 FFPE Tissue LEV DNA Purification Kit. DNA was exome-captured and amplified with Agilent SureSelect XT (G9611B) and capture library (5190-8881). WES was performed using the Illumina HiSeq 2500 or NextSeq 500 platform with multiple samples per lane using 2 × 100 paired-end chemistry. RNA was isolated from the same sections with QIAGEN AllPrep FFPE, and libraries were prepared with Illumina TruSeq Stranded with RiboZero Gold (RS-122-2301). mRNA-Seq libraries were run at 2 samples per lane on an Illumina HiSeq2500 sequencer in high-output mode using 2 × 50 paired-end chemistry.

WES was performed on FFPE tumor tissue, with peripheral blood mononuclear cells serving as a matched normal. Library preparation was performed with the SureSelect XT Human All Exon V6 + UTR kit (Agilent, Santa Clara, CA, USA), and pooled samples were sequenced on the HiSeq2500 platform (Illumina). The resulting somatic and germline WES sequencing files were aligned to Hg38 using bwa (v0.7.17), sorted, and indexed, and duplicates were marked using biobambam2 (v2.0.87). BAMs were re-aligned with Abra2 (v2.22), followed by somatic and germline variant detection with Strelka2 (v2.9.10), Cadabra (from Abra2 v2.22), and Mutect2 (GATK v4.1.4.0). The capture of exonic sequences was verified using the Picard (v2.21.1) CollectHsMetrics tool, and the quality of sequencing data was verified using FastQC (v0.11.8) and the Picard suite’s CollectAlignmentSummaryMetrics, CollectInsertSizeMetrics, QualityScoreDistribution, and MeanQualityByCycle tools. Variants with matched normals were filtered by the following criteria: protein-coding mutations only, Cadabra indel quality >10.5, Mutect2 indel quality >6.8, or (single-nucleotide variant) SNV quality >9.2, Strelka2 indel quality >15.2 or SNV quality >19.7. Additionally, Cadabra indels with quality <35 required a supporting high-quality call from either Strelka2 or Mutect2, and Strelka2 calls with SomaticEVS <20 similarly required a matching call from either Mutect2 or Cadabra. Variants for tumor-only samples were detected by Mutect2 and filtered to retain the protein-coding mutations. The remaining variants required at least 5 supporting reads and a minimum read depth of 40 or 10 supporting reads and a minimum read depth of 80 if MAF <5%. The variants with a MAF >5% in normal tissue were dropped, as were the variants appearing at rates above 1% in any subpopulation in either GnomAD or 1000 Genomes databases. To counter FFPE artifacts, C>T and G>A substitutions required a minimum MAF of 10%. Tumor mutational burden (TMB) was calculated from small indels and substitutions identified by WES and divided by the megabases adequately covered by sequencing reads. Whole-exome sequencing data for both tumor and germline was available for 28 samples (representing 22 unique patients) at baseline. Tumor-only whole-exome sequencing data—without matched normal—was available for a further 2 samples (representing 2 patients), for a total of 30 samples across 24 patients with WES data. Oncoplots were created in R using maftools v2.10.0, with variant genes limited to those implicated in breast cancer from the COSMIC Cancer Gene Census Tier 1 list (https://cancer.sanger.ac.uk/cosmic).

### Copy Number Variation and Subclonal Heterogeneity Analysis

LCCC1419 patients with matched DNA normal and DNA tumor samples (*n* = 22) were processed through trim galore v. 0.6.2. The resulting trimmed FASTQs were aligned to the human reference genome FASTA file Homo_sapiens_assembly38.fasta (from the GATK hg38 file bundle) with the bwa mem command from BWA v. 0.7.17 with default parameters. The resulting SAM files were sorted, converted to BAM files, and indexed using SAMtools v. 1.9. The matched samples from each patient were then processed through the Sequenza v. 3.0.0 workflow (gc_wiggle with window size of 50 basepairs, bam2seqz, seqz_binning, sequenza.extract, sequenza.fit, and sequenza.results all with default parameters). The resulting patient-level segment files were then modified to conform to the format required by GISTIC. This conversion was performed with standard BASH scripting and included a log2(x) - 1 transformation of Sequenza’s raw depth ratio value for GISTIC. The resulting modified segment files were then concatenated across samples and ran through GISTIC v. 2.0.23 using the hg38.UCSC.add_miR.160920.refgene.mat file (from docker://shixiangwang/gistic:1.2) for the -refgene parameter. CNVKit was run using the “batch” mode for primary tumors and BrM separately (with their matched normal samples, respectively). All normal samples within each group were pooled together to generate a pan-sample normal control. Agilent’s SureSelect Human All Exon V6+UTRs probed bed file was provided for the –targets parameter, a standard hg38 refFlat.txt file was provided for annotations, and a k50 umap mappability BED file was provided for the –access parameter. Outputs from Sequenza were paired with each patient’s corresponding MuTect2 somatic variant calls to create PyClone-VI input files using Sequenza copy number information and MuTect2’s variant read support information. Files were created for each primary tumor and BrM sample separately, and paired input files were created for patients that had both primary tumor and BrM data available. The resulting files were run through PyClone-VI using default parameters.

### Neoantigen Prediction Using Genomics Data

Tumor antigens were predicted from a comprehensive set of genomic sources (single-nucleotide variations, insertions/deletions, gene fusions, alternative splice variants, cancer testis antigens, overexpressed self-antigens, and viral and endogenous retroviral antigens) using methods developed by our group and implementations of methods developed by others (14–22). Briefly, whole-exome sequencing was used to identify tumor-specific genetic variants (single-nucleotide variations, insertions/deletions, gene fusions), and RNA sequencing was used to confirm the expression of these variants. RNA sequencing data alone were used to evaluate for expressed alternative splice variants, viral and endogenous retroviral antigens. Cancer testis antigens/overexpressed self-antigens were evaluated using RNAseq data, but WES data was used to incorporate germline variants. RNA sequencing data was also used to infer tumor MHC haplotypes *via* HLAProfiler, the most accurate tool for MHC haplotype inference (23). Peptide fragments generated *in silico* are evaluated for predicted binding affinity to tumor MHC alleles using NetMHCpan-4.1 (24). Peptides with predicted binding affinity <500 nM were considered positive binders (*e*.*g*., potential tumor antigens), while peptides with predicted binding affinity <50 nM considered strong binders and more likely to be tumor antigens (25).

### RNA-Seq Data Processing

RNA-Seq Paired FASTQs were run through trim galore v. 0.6.2 using –paired parameter. STAR v. 2.7.0f was used to index the reference genome Homo_sapiens.GRCh38.dna_sm.primary assembly.fa from GATK and to map trimmed reads to reference (using parameters –quantMode TranscriptomeSAM – outSAMtype BAM SortedByCoordinate –sjdbGTFfile Homo_sapiens.GRCh38.100.gtf). Gffread v. 0.11.7 was used to create a transcriptome reference using the reference genome and the gtf file Homo_sapiens.GRCh38.100.gtf. The “toTranscriptome” alignments from STAR were used with Salmon v. 1.1.0 using “salmon quant -l a”. Sample quality was assessed using MultiQC v1.9 with Picard CollectRnaSeqMetrics, and samples with less than 20 M coding reads were excluded as this threshold has been found to approximate the minimal sequencing depth to achieve equivalent detection to microarrays (26). Counts were log2-transformed and the upper quartile normalized for further downstream analysis. Some patients’ tumors were sequenced multiple times (technical replicates), and in such cases, gene-level expression values were averaged across technical replicates.

### Intrinsic Subtype Analysis

Intrinsic subtype analysis was performed according to the methods described in Picornell et al (27). The R package heatmaply (28) was used for heat map visualization with hierarchical clustering based on average linkage.

### Immune Gene Signature Expression Analysis

Thirty-two immune gene signatures were chosen to reflect the diversity of tumor-infiltrating immune cell populations and to minimize redundancy (refer to associated.gmt file in the **Supplementary Material**). The *binfotron* R package (29) was used to compute the differential gene expression [along with the DESeq2 dependency (30)], produce a volcano plot, and calculate immune signature metagene scores (median log2 expression values) for downstream analysis. ssGSEA was performed using the R packages *GSVA* and *GSEABase* (31, 32). CIBERSORTx immune cell fraction imputation using the LM22 matrix was also performed (33).

### TCR/BCR Repertoire Analysis

Immune chain inference was performed on RNA-Seq samples *via* MiXCR 2.1.2 for TCR chains (34) and V’DJer 0.12 (35) for BCR chains. The consensus BCR contigs from V’DJer were quantified using Salmon 0.13.11 (36). Repertoire diversity was calculated using a model-based approach, which improves estimations of diversity in part by minimizing known sources of estimate bias (37).

### Survival Analyses

Multivariable Cox proportional hazards modeling was performed using the *survival (* 38 *)* R package. The model included cancer stage, age at primary tumor diagnosis, and race. Time from initial diagnosis, from diagnosis of any metastasis regardless of anatomical location, or from diagnosis of BrM to an event was interrogated. Hazard ratios and 95% confidence intervals were returned for gene signature covariates and visualized using the *forestplot (* 39) R package.

### Accession Numbers and Data Sharing

Sample information for RNA-seq and DNA-seq fastQ runs, including the clinical information, were uploaded to the NCBI’s dbGaP repository (accession no. phs002457.v1.p1) and SRA.

## Results

### Patient Cohort Characteristics

Tissues and blood from patients with clinical TNBC (*n* = 25) were included in this analysis, including BrM tissues (*n* = 19), primary breast tumors (*n* = 17), and whole blood samples (*n* = 22). The specimen numbers by tissue type and analysis (IHC, RNA, or DNA) are outlined in **Supplementary Figure S1**. Patient demographics are included in **Table 1**, with individual clinical–pathological characteristics and specimen availability presented in **Supplementary Table S1**. The majority of patients were Caucasian (*n* = 17, 68%), with African American, Asian, and other ethnicities represented [*n* = 6 (24%), *n* = 1 (4%), and *n* = 1 (4%), respectively]. One male was included in the cohort. Median age at breast cancer diagnosis was 46.7 years (range, 29–70.9), while median age at BrM diagnosis was 51.5 years (range, 31.7–72). The majority (*n* = 12, 48%) of patients were initially diagnosed with stage II disease prior to recurrence; a minority (*n* = 2, 8%) were diagnosed with *de novo* stage IV TNBC. In addition to BrM, other sites of disease included the liver (*n* = 8, 32%), bone (*n* = 15, 60%), lung (*n* = 16, 64%), and non-local lymph nodes (*n* = 18, 72%). Ten patients (40%) were initially diagnosed with a solitary BrM, while 7 patients were diagnosed with 5 or greater BrM (28%). BrM was supratentorial in *n* = 23 (92%) patients and infratentorial in *n* = 13 (52%) patients. The median progression-free survival (*e*.*g*., time from primary TNBC diagnosis to the diagnosis of any metastasis) was 1.8 years (range, 0–19.5). The median OS from primary TNBC diagnosis was 3.7 years (range, 0.9–19.8), while the median OS from BrM diagnosis was 1.2 years (range, 0 – 8.9).

**Table 1:** Relevant demographic, subtype, and clinical diagnostic information for the LCCC1419 TNBC cohort.

Characteristics	Number (%)
Demographic information	
Means of enrollment (*n* = 25)	
LCCC 1419 consent Health Registry consent Waiver of consent	6 (24%) 16 (64%) 3 (12%)
Race (*n* = 25)	
African American Asian Caucasian Other	6 (24%) 1 (4%) 17 (68%) 1 (4%)
Ethnicity (*n* = 25)	
Hispanic Not Hispanic Unknown	2 (8%) 22 (88%) 1 (4%)
Sex (*n* = 25)	
Female Male	24 (96%) 1 (4%)
Smoking status (*n* = 25)	
Never smoker Current smoker Former smoker	14 (56%) 4 (1%) 7 (28%)
Subtype information	
Subtypes by primary resection (*n* = 25)	
Luminal A (ER/PR+, HER2-) Luminal B (ER/PR+, HER2+) HER2 (ER-, PR-, HER2+) TNBC (ER-, PR-, HER2-) Mixed (two primaries tested with different results) Unknown	1 (4%) 0 (0%) 0 (0%) 23 (92%) 0 (0%) 1 (4%)
Subtypes by CNS resection (*n* = 21)	
Luminal A (ER/PR+, HER2-) Luminal B (ER/PR+, HER2+) HER2 (ER-, PR-, HER2+) TNBC (ER-, PR-, HER2-) Radiation necrosis Unknown	1 (5%) 0 (0%) 0 (0%) 15 (71%) 1 (5%) 4 (19%)

### Intrinsic Subtype Classification of Primary TNBC and Brain Metastases

Intrinsic molecular subtype analysis using RNAseq data illustrated that the majority of clinically determined TNBC samples were of the basal subtype (**Supplementary Figure S2**). Of the BrM (*n* = 19), 16 were classified as basal (84%), with normal-like (*n* = 2) and HER2-enriched (*n* = 1) comprising a small fraction of the cohort. Similarly, primary tumors (*n* = 15) were predominantly classified as basal (*n* = 13, 87%), with the remaining tumors being normal-like (*n* = 2). Notably, the 4 samples that were called normal-like by PAM50 analysis had a basal subtype as the second highest identity probability. There were 2 cases with discordant receptor classification between primary tumor and BrM by immunohistochemistry (*n* = 1 ER+/PR+/HER2- Luminal A primary converted to a TNBC BrM, and *n* = 1 TNBC primary converted to an ER+/PR+/HER2- Luminal A BrM) (**Table 1**; **Supplementary Figure S2**). Despite the potential subtype switching between primary and BrM, these samples were included in the downstream analyses.

### Mutational, Somatic Copy Number Alteration, and Subclonal Analyses of Primary TNBC Tumors and Brain Metastases

**Figure 1:**
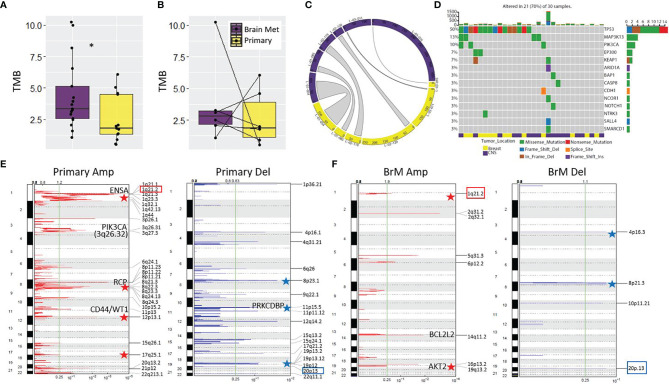
Mutational analysis of the LCCC1419 cohort. **(A)** Increased tumor mutational burden (TMB) was observed for BrM (*n* = 17) relative to primary (*n* = 13); *p <0.05, Wilcoxon rank-sum test. **(B)** No difference was observed between TMB for matched pairs (*n* = 6). **(C)** Circos plot showing the total number of variants and the proportion of variants shared between matched primary/BrM (*n* = 6). **(D)** Oncoprint displaying the mutational spectrum of cancer-associated genes with known etiology to breast cancer (COSMIC Tier 1, Sanger Institute). Representation of the somatic copy number alterations in **(E)** primary TNBC and **(F)** BrM as determined by CNVkit/GISTIC 2.0 analysis. Significant amplicons (Amp) or deleted (Del) regions are annotated (*q <*0.25, green line). The 1q21.2 amplification and the 20p13 deletion, which are shared genomic features of primary and BrM TNBC, are highlighted by colored boxes. Relevant oncogenes and tumor suppressors are annotated on the plots, and stars indicate genomic regions where copy number alterations are known to contribute to breast cancer/aggressive basal breast cancer.

First, we analyzed the tumor mutational burden (TMB) of primary tumors (*n* = 13) relative to BrM (*n* = 17) using WES. On average, BrM harbored a greater mutational load than primary tumors (median 3.33 *vs*. 1.78 mutations/Mb, respectively, *p* < 0.05; **Figure 1A**). Upon analysis of matched primary-BrM WES pairs (*n* = 6), however, there was no significant difference between tumor location and TMB (median 2.80 *vs*. 1.88 mutations/Mb, respectively, *p* = 0.69; **Figure 1B**). An analysis of shared mutations within matched pairs revealed varying degrees of mutational conservation between anatomical locations (**Figure 1C**). The degree of variant sharing between matched pairs (**Figure 1C**) was generally greater than the degree of mutations shared between primaries and BrM globally (**Supplementary Figure S3**), highlighting interpatient tumor heterogeneity and mutational divergence. We assessed whether a survival benefit was conferred by increasing TMB, as TMB has been considered a proxy for neoantigen burden (40, 41). There was no significant association between TMB and survival (*p* = 0.07) from the time of primary TNBC diagnosis in the context of a model that included standard clinicopathological features (age at diagnosis of primary tumor, stage, and race) **(** **Supplementary Figure S4** **)**. Next, we examined the mutational spectrum of genes with known associations to breast cancer development (42). We found that these genes were altered in 70% of combined primary and BrM samples, with *TP53* being the most commonly mutated gene (mutated in 50% of samples, *n* = 15), in accordance with its known relevance to TNBC (43) (**Figure 1D**). The next most frequently altered genes were *MAP3K13* and *PIK3CA*, which were mutated in 13% (*n* = 4) and 10% (*n* = 3) of samples, respectively; all other genes were mutated less frequently, occurring in 7% or less (*n* ≤ 2) of samples (**Figure 1D**).

We next analyzed the recurrent copy number alteration patterns in primary and BrM samples using Sequenza/GISTIC 2.0 (44, 45). The primary TNBC samples harbored 3 significant amplicons and 1 deleted region, whereas BrM was more profoundly altered with 15 significant amplicons and 12 regions of deletion (*q* < 0.25; **Supplementary Figure S5**). At this level of genomic resolution, only 2 regions were commonly altered between primary and BrM (11p13 amplicon and 13q11 deletion). In breast cancer, these two sites are previously known to be amplified or deleted, respectively (46, 47). Interestingly, a number of amplicons/deleted regions identified in this cohort are known to be associated with breast cancer/aggressive basal breast cancer, such as gains of 1q, 8p11-12, 8q, 12p13, 13q34, 17q, and 19q and deletions of 3p, 4p16.3, 8p, 11p15, 17p, and 19p13 (48–58). Documented breast cancer oncogenes (*NOTCH2*, *ENSA*, *PIK3CA*, *CD44*, *WT1*, *BCL2L2*, *AKT2*, and *TFF3*) and tumor suppressors (*BRCA2* and *PRKCDBP*) reside or are in close proximity to some of these significantly amplified/deleted genomic regions, and these alterations potentially contribute to TNBC progression and BrM development.

Since somatic copy number alteration (SCNA) detection tools are prone to high false positive rates as well as issues with precision and accuracy (59, 60), we also performed SCNA calling using CNVkit (61), which combines all normal samples into a pooled reference to increase performance. In contrast to Sequenza, this method identified a greater number of SCNA in primary TNBC relative to BrM, showing that, as a collective group, primary TNBC samples harbored 27 significant amplicons and 17 deleted regions, whereas BrM had 8 significant amplicons and 4 regions of deletion (*q* < 0.25; **Figures 1E, F** **)**. Despite notable differences between the two methods, there was a concordance in the results as well, with corroboration of 8p11.22 and 11p13 amplification in primary TNBC and validation of 14q11.2 and 19q13.2 amplification and 4p16.3 deletion in BrM (**Figures 1E, F** **;** **Supplementary Figure S5**). The CNVkit SCNA analysis also highlights the potential importance of oncogenes (*e*.*g*., RCP, CD44, WT1, BCL2L2, and AKT2) and tumor suppressors (*e*.*g*., PRKCDBP) to TNBC etiology and metastatic progression, as significant amplicons/deleted regions harbor these genes.

Finally, we examined the ploidy, tumor purity, and subclonal makeup of tumors in this cohort. No differences in cellular ploidy were noted between primary TNBC and BrM (median ploidy of 3.2 and 3.25, respectively; **Supplementary Figure S6A**). Similarly, no significant differences in tumor purity were observed between groups (median purity of 0.57 and 0.76, respectively; **Supplementary Figure S6B**). An analysis of subclonal tumoral architecture using Pyclone-VI (62) showed no difference between the number of clones per tumor in each location, where a median of 3.5 clones per tumor in primary TNBC (range, 1–5 clones/tumor) and a median of 4 clones per tumor in BrM (range, 3–6 clones/tumor) were observed (**Supplementary Figure S6C**). A subclonal assessment in patients with matched primary/BrM pairs showed that some pairs had similar clonal constituency between anatomical sites (*e*.*g*., patients L-01-054, L-01-085, L-02-119, and L-03-016), whereas other pairs showed signs of divergent clonal evolution (*e*.*g*., L-02-120 and L-03-011) (**Supplementary Figure S6D**). Interestingly, 5 of 6 matched pairs the dominant subclone harbored the highest mutational burden, which was reflected in the analysis of unmatched tumors as well (not shown), suggesting that increased mutational load may endow these subclones with a selective growth advantage.

### Tumor Antigen Landscape

**Figure 2:**
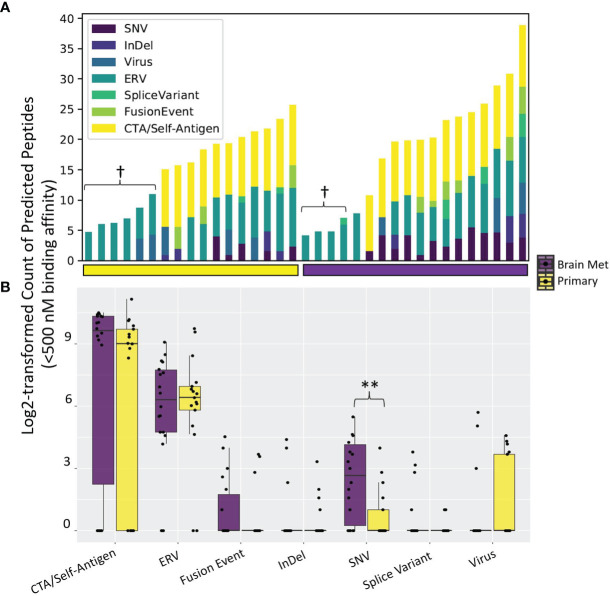
Tumor antigen sources among the LCCC1419 patients. A comprehensive bioinformatics prediction pipeline that exports **(A)** the number of neoantigen-derived MHC class I-binding peptides (Kd < 500 nM) broken down by antigen source was employed. Some patients’ tumors did not have associated tumor or normal whole-exome sequencing data, and as such, antigen sources that require DNA sequencing data (single-nucleotide variants, InDels, cancer testis antigens, or fusion events) are not able to be queried in these cases (denoted by †). **(B)** Distribution of the number of neoantigen-derived MHC class I-binding peptides (Kd < 500 nM) broken down by antigen source, corresponding to **(A)**; **p <0.01 (Wilcoxon rank-sum test).

We next performed a comprehensive analysis of the neoantigen landscape in this cohort. We queried a range of neoantigen sources, including single-nucleotide variants (SNVs), insertion/deletion events (InDels), splice variants, structural fusion events, cancer testis antigens (CTAs)/self-antigens, endogenous retroviruses (ERVs), and viral sources excluding ERVs (63) (**Figure 2**). The predominant antigen sources in both primary TNBC and BrM were CTAs/self-antigens and ERVs (**Figures 2A, B** **)**. Upon comparison of the number of predicted neoantigen-derived peptides, there were significantly more SNV-derived MHC class I-binding peptides in BrM as compared to primary TNBC (*p* = 0.005), with no differences seen between groups with respect to other neoantigen sources (**Figure 2B**). This analysis together shows that TNBC harbors a diverse set of potentially therapeutically actionable neoantigen-derived peptides.

### Comparison of Immune Gene Signatures Between Primary TNBC and BrM

**Figure 3:**
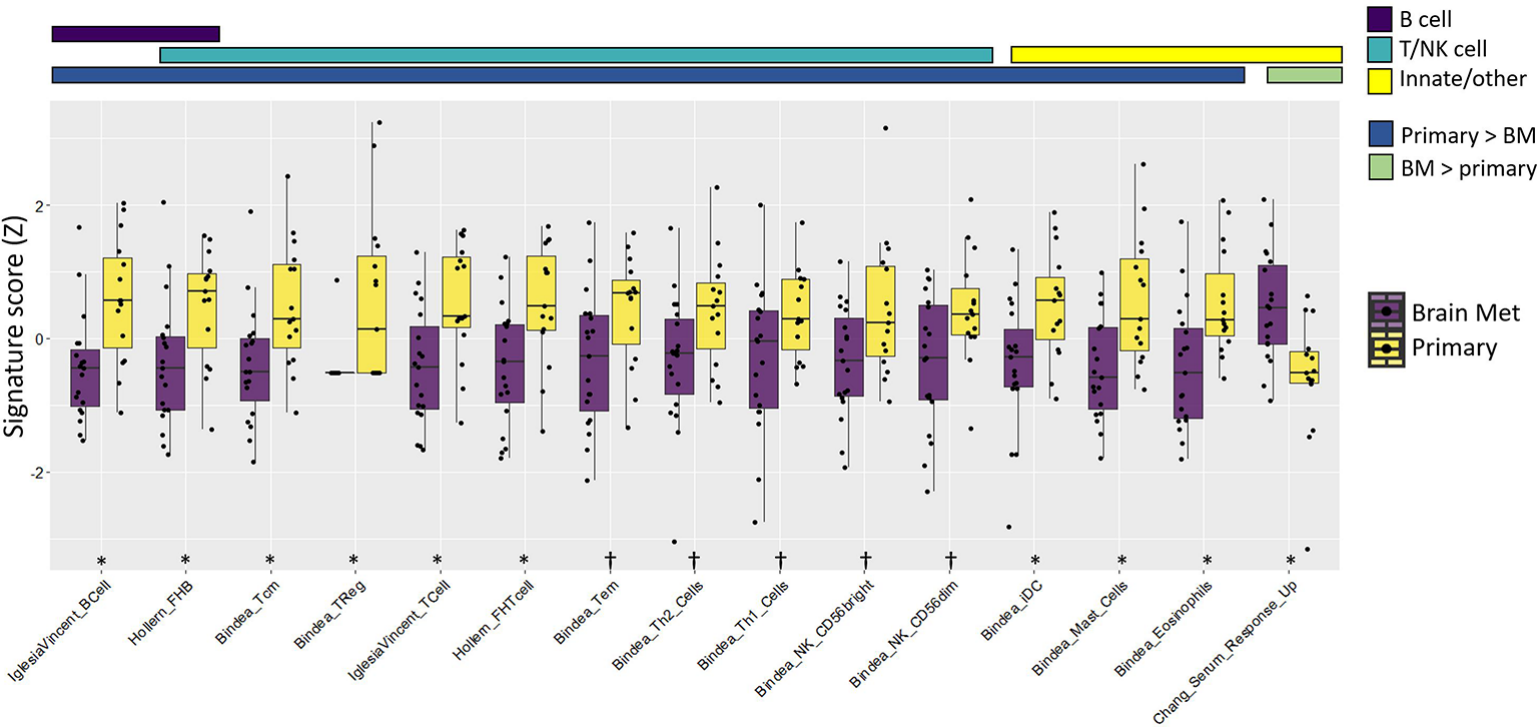
Immune gene signature metagene analysis showed an overall immune cell deficit in BrM relative to primary triple-negative breast cancer. The colored bars above the plot indicate both the immune cell category assigned to the respective signatures and whether the signatures were increased/decreased in the primary tumor relative to BrM. Wilcoxon rank-sum test was performed on Z-transformed signature scores to determine the statistical significance after false discovery rate correction. Significance codes: ^†^ *q* < 0.1, *q < 0.05.

Immune gene signatures (IGS) representing multiple components of the immune system, including B cells, T cells, natural killer cells, and innate immune cells along with immune cell phenotype frequencies, were evaluated between primary TNBC tumors (*n* = 15) and BrM (*n* = 19) using RNA-Seq (**Figure 3**; **Supplementary Figure S7**; **Supplementary Data S1**). The majority of IGS across each of these categories were lower in TNBC BrM compared to primary TNBC. A gene signature associated with fibroblast-associated wound healing [Chang_Serum_Response_Up (64)] was significantly higher in BrM relative to primary tumors (*q* < 0.05). RNAseq expression data from primary tumors and BrM were also assessed using CIBERSORTx (65) to determine relative frequencies of 22 immune cell subtypes (LM22) to tumor composition. In this analysis, naïve B cells and M1 macrophages were lower in BrM compared to primary tumors, while eosinophils and neutrophils were higher in BrM tissues (*q* < 0.05) (**Supplementary Figure S8**). The expression of the 20-gene immunologic constant of rejection signature [ICR (66)], which is representative of Th1-mediated immunity, cytotoxic function, and tissue-specific destruction (*e*.*g*., GVHD, autoimmunity, and allograft rejection), was also significantly reduced in BrM relative to primary TNBC (**Supplementary Figure S9A**). Additionally, the blood transcriptional modules reported by Rinchai et al. (67) were queried against our dataset and showed a significant reduction of B and T cell modules in BrM relative to primary TNBC (**Supplementary Figure S9B**), concordant with the abovementioned data. These results are together consistent with an overall immune-excluded brain tumor microenvironment (TME) in the context of TNBC BrM.

### T and B Cell Repertoire Analysis

**Figure 4:**
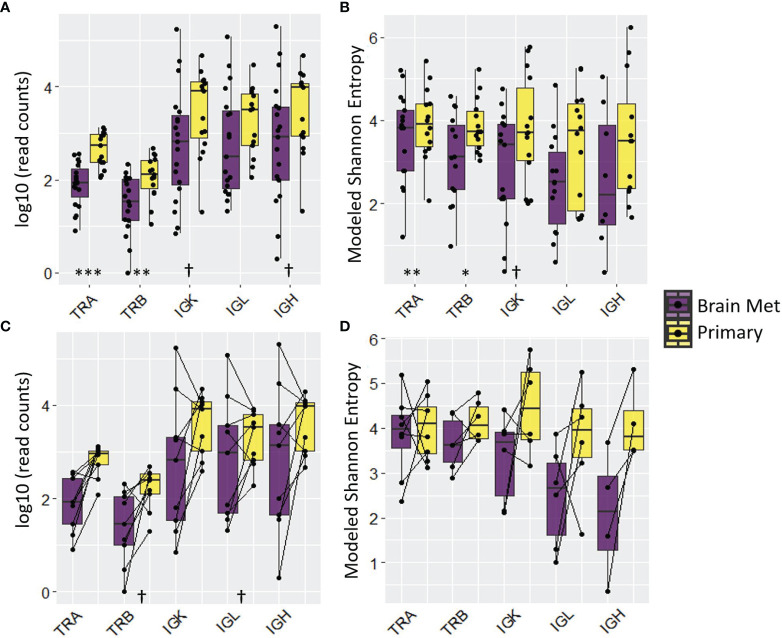
T cell and B cell repertoire analysis revealed adaptive immune cell deficit in BrM relative to primary triple-negative breast cancer. The distribution of read counts and modeled Shannon entropy for all samples is displayed in **(A, B)**, respectively. The same information is displayed, respectively, in **(C, D)** for matched pairs (note that the number of matched pairs varies due to the presence/absence of relevant reads). Wilcoxon rank-sum test was performed on log_10_-transformed (read counts) or raw (modeled Shannon entropy) values to determine the statistical significance. Significance codes: ^†^ *p* < 0.1; *p < 0.05; **p < 0.01; ***p < 0.001.

We used RNA-Seq data from primary TNBC tumors (*n* = 15) and BrM (*n* = 19) to perform T cell and B cell repertoire (TCR/BCR) profiling. Relative to primary TNBC, TNBC BrM had lower read counts of T cell receptor alpha and beta (TRA, *p* < 0.001 and TRB, *p* < 0.01), with BCR heavy chain and light chain abundance showing trending but non-significant differences (**Figure 4A** **)**. This result is in accordance with RNA-seq data that showed less T cell and B cell abundance in the primary samples relative to BrM (**Figure 3**). Repertoire diversity was indexed as modeled Shannon entropy (37), which is a diversity index that accounts for both the *richness* of the sample (*e*.*g*., the number of unique TCR/BCR sequences) and relative species *abundance* (evenness) (68, 69). Thus, a large Shannon entropy score reflects a more diverse distribution of TCR/BCR sequences. The modeled Shannon entropy (TCR/BCR diversity) was lower for BrM compared to primary tumors (TRA, *p* < 0.01 and TRB, *p* < 0.05) (**Figure 4B**). A comparison of matched BrM and primary TNBC pairs *only*, however, did not show a reduction of TCR/BCR read counts and modeled Shannon entropy (**Figures 4C, D** **)**.

### Differential Gene Expression and Pathway Analysis Support an Immune Cell Deficit in TNBC BrM Relative to Primary TNBC Tumors

**Figure 5:**
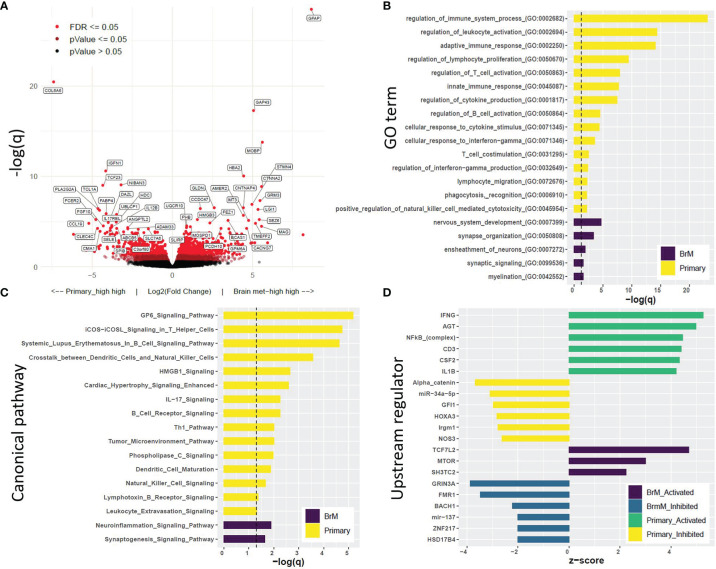
Differential gene expression analysis and Gene Set Enrichment Analysis further support the immune cell deficit in BrM relative to primary triple-negative breast cancer (TNBC). **(A)** Volcano plot displaying differentially expressed genes (DEGs) in primary TNBC relative to BrM, with the legend showing color-coded levels of significance. **(B)** Top Gene Ontology terms associated with DEGs. DEGs with LFC > |1| and *q* < 0.1 (equating to 468 genes for primary_vs_BrM and 463 genes for BrM_vs_primary) were subjected to PANTHER overrepresentation test (dotted line represents *q* = 0.1). **(C)** Canonical pathway analysis (Ingenuity Pathway Analysis, IPA) of DEGs. The pathways displayed were significant at *q <*0.05 (dotted line) and were associated with a significant *z*-score (*z* >|2|) which indicates associative activity. **(D)** Upstream regulator analysis (IPA) displaying top regulators (*z* >|2|, *q* < 0.1) identified to be associated with an active or inhibited state in primary *versus* BrM TNBC [see **Supplementary Data S2, S3** for the full list of DEGs (*q* < 0.1) and upstream regulators (*z* >|2|, *q* <0.1).

Gene expression was evaluated by utilizing RNA-Seq data between the primary tumor (*n* = 15) and BrM (*n* = 19) tissues. In total, there were 1,669 differentially expressed genes (DEGs) between these 2 groups, with 935 genes upregulated and 734 genes downregulated in the BrM tissues compared to primary tumors (*q* ≤ 0.1; **Figure 5A**; **Supplementary Data 2**). Gene ontology (GO) analysis of DEGs revealed a significant enrichment of immune-related terms in the primary TNBC tumors compared to the BrM (particularly terms reflecting adaptive immune system involvement), whereas GO terms associated with the nervous system were significantly higher in the BrM relative to the primary TNBC tumors (**Figure 5B**). Canonical pathway analysis (Ingenuity Pathway Analysis, IPA) of DEGs in primary tumors *versus* BrM illustrated a similar preponderance of immune signaling-related pathways as well as nervous system-related pathways associated with DEGs in BrM relative to primary tumors (**Figure 5C**). Upstream regulator analysis (IPA) further demonstrated an association of immune-related signaling activity with DEGs in primary tumors relative to BrM (*e*.*g*., IFNG, NFKB, CD3, CSF2, and IL-1β) and an association of potential oncogenic drivers (*e*.*g*., TCF7L2, mTOR, and SH3TC2) with regulation of BrM DEGs (**Figure 5D**; **Supplementary Data S3**).

### Adaptive Immune Cell Signatures Are Associated With Improved Survival for Patients With TNBC BrM

We examined the survival association of standard clinicopathological variables (age at diagnosis of primary tumor, stage, and race) with different time metrics to event: (1) time from diagnosis of primary TNBC to death, (2) time from diagnosis of any metastatic disease to death, and (3) time from diagnosis of BrM to death. Of these variables, only older age was significantly associated with poor survival using each of these time metrics (**Supplementary Figure S10**), which was similar to other recent reports (70, 71). Next, survival associations relative to IGS expression were evaluated using multivariable CoxPH models in both primary TNBC and BrM. The IGS features in primary TNBC tumors which were associated with improved survival following metastatic diagnosis included T cell, B cell, and dendritic cell (DC) signatures (**Supplementary Figure S11A**). Interestingly, a fibroblast serum response/wound healing signature (64) (“Chang_Serum_Response_Up”) was associated with a significantly poorer survival (*p* = 0.025) in BrM (**Supplementary Figure S11B**) after a diagnosis of metastasis.

## Discussion

In this study, we examined the genomic and transcriptomic landscape of TNBC BrM and primary tumors to further the understanding of TNBC BrM etiology and the tumor immune microenvironment. Despite recent progress in the treatment of ER+ and HER2+ BCBrM with newer brain-penetrant, targeted therapies, the treatment options for TNBC BrM remain largely restricted to chemotherapy and local therapy due to lack of known targets. A growing appreciation for the role of immunotherapy in the treatment of TNBC highlights the need to better understand the immune context of BrM as we consider incorporation of immunotherapy into the care of our patients (72).

Through whole-exome sequencing, we report that BrM, as a group, exhibited a greater TMB than primary tumors, though this observation was not recapitulated in matched tissue pairs. We suspect that this was due to underpowering of our study to assess matched pairs (*n =* 6) in the context of TNBC tumor biological heterogeneity (73). An analysis of shared variants showed that matched primary TNBC and BrM samples were more alike than inter-patient primaries and inter-patient BrM, showing that TNBC is a heterogeneous disease with potentially non-redundant mechanisms of tumorigenesis. A subclonal analysis of matched pairs also showed that some patients displayed patterns of divergent evolution between primary tumors and BrM. The mutational spectrum of genes with known causality to breast cancer tumorigenesis was also queried. *TP53* was commonly mutated in this cohort (50%), supporting its causal role in the development of TNBC, while other genes such as *MAP3K13* and *PIK3CA* were mutated at a lower frequency.

Copy number variation analysis revealed common and unique genomic alteration events between primary TNBC and BrM. 11p13 was commonly amplified in both primary tumors and BrM. This genomic location harbors the *CD44* gene, which is used to discern breast CSCs, although it has been shown that it is not likely a driver of amplification of this region in basal breast cancer (74). *WT1*, which also resides at 11p13, has been shown to promote a mesenchymal phenotype in breast cancer cells as well as to elicit resistance to taxane therapy (47). Regions of 1q were also commonly amplified, which supports a known role for this genomic location in breast cancer development (48). The amplification and increased expression of ENSA (1q21.3) have recently been shown to drive TNBC progression *via* positive regulation of cholesterol biosynthesis (58). 13q11 was deleted in both primary and BrM TNBC, and this site is proximal to *BRCA2* (located on 13q13.1). Whether or not the loss of 13q11 has any *BRCA*2-regulatory functionality is unknown, although deletions in 13q and 14q are common in *BRCA2*-mutated breast cancers (75). Common deletion of 20p13 was also observed. While this location is known to be deleted in colon cancer (76), its association with breast cancer has yet to be explored. Other common deletions identified included 1p36 and 11p15.5. In ductal breast carcinoma, the 1p36 deletion is associated with grade, *ERBB2* loss, and loss of BCL2 expression (57) and is known to be a common feature underlying breast cancer development and the carcinogenesis of various cancer types (77). BrM-specific deletion at 11p15.5 (region harboring *PRKCDBP*) was also observed, and the chromosomal loss of this region is associated with BCBrM, with *PRKCDBP* identified as a putative tumor suppressor (53).

In primary TNBC, notable amplicons were associated with both arms of chromosomes 1 and 8. These locations are associated with breast cancer cytogenetics and pathology (48, 78, 79) and harbor genes (*NOTCH2* and *RCP*, respectively) associated with breast cancer etiology (49, 80). There were also several notable alterations specific to BrM. Regions harboring the oncogenes *BCL2L2* (14q11.2), *AKT2* (19q13.2), and *TFF3* (21q22.3) were amplified in BrM. BCL2L2 is an anti-apoptotic protein that has an oncogenic role in many solid tumor types, and it has been found to contribute to breast cancer progression through its upregulation *via* hypermethylation of the negative-regulatory miR-129-2 (81). While *PIK3CA* was only mutated in 10% of evaluated samples in this study, *AKT2* upregulation *via* genomic amplification may have a significant impact on TNBC BrM progression. Dysregulation of the PI3K/AKT/mTOR axis is a common feature of TNBC (82), and this pathway represents a promising target in this disease context. *TFF3* is also associated with breast cancer metastasis, where its expression predicts poor survival (83), and it is also associated with residual invasive disease following neoadjuvant chemotherapy in breast carcinoma (84). Interestingly, *TFF3* was also found to be significantly upregulated in T cell-cold tumors of diverse tissue types, and it was in the top percentile of genes differentially expressed in T cell-cold *versus* T cell-hot breast cancers (85), which suggests its potential as an immunotherapy target. These mutational and copy number analyses together highlight potential causative genomic alterations contributing to TNBC progression and BrM.

A systematic evaluation of the neoantigen landscape in LCCC1419 was undertaken here. Using a suite of bioinformatics prediction software, we analyzed tumor-associated antigens (*e*.*g*., CTAs/self-antigens), traditional tumor-specific antigens (TSAs; *e*.*g*., SNVs), and alternative TSAs [*e*.*g*., derived from splice variants, chromosomal structural variants, InDels, ERVs, and other viral antigens (63)]. We found that both primary TNBC and BrM harbored substantial numbers of high-affinity MHC class I-binding peptides derived from CTAs and ERVs relative to other antigen sources. CTAs are known to be associated with aggressive hormone-negative breast cancers and poor survival; however, they have also been associated with robust immunogenicity in some contexts (86). ERVs, which are evolutionary remnants of viral insertional mutagenesis, are also potentially powerful immunogens (18). Although ERV transcriptional regulation is often epigenetically silenced in normal cells, tumor cell-specific derepression is known to occur and is associated with a response to immune checkpoint blockade (ICB) in multiple cancer types (18, 87, 88). As such, antigens derived from CTAs and ERVs may be invaluable immunotherapeutic targets for vaccine strategies targeting TNBC and BrM lesions. Relative to primary TNBC, we also found an elevated SNV mutational load associated with BrM. This augmented TSA burden in BrM also represents a potential vulnerability to be targeted by combination immunotherapeutic approaches, including neoantigen vaccine strategies.

A comprehensive analysis of transcriptomic data derived from this cohort was performed to further understand the difference between the tumor immune microenvironment of primary and BrM TNBC. We found that BrM lesions harbored significantly less immune infiltrate than primary tumors. This is not surprising, as the brain has historically been considered an immunologically protected organ (89). A recent study with RNA array data in BCBrM, agnostic to subtype of BC, has similarly reported reduced immune scores in BCBrM relative to primary tumors (90). The general dearth of immune involvement in the BrM spanned both adaptive (T and B cell) and innate (DC, eosinophils, and mast cells) immune populations, indicative of a broad immune deficit relative to primary tumors and again similar to recent reports (90). Interestingly, BrM displayed an elevated expression level of genes involved in a serum-induced fibroblast wound healing response (64). This finding may suggest that, relative to primary TNBC, BrM lesions are more reliant on aberrant wound healing properties, requiring increased levels of stromal involvement for growth and maintenance, as seminally put forth by Dvorak (91). We also observed that BrM had significantly decreased TCR (TRA/TRB) abundance and diversity as compared to primary tumors, and this association was verging on significance for certain immunoglobulin classes. These metrics are important, as increased TCR abundance and diversity have been associated with a response to ICB in multiple solid tumor types (92). DEGs between primary and BrM TNBC also reflected a BrM-specific immune deficit. Gene Ontology and canonical pathway analysis showed that genes that exhibited relatively lower expression levels in BrM *versus* primary tumors were enriched for terms related primarily to an adaptive immune response. An upstream regulator analysis further supported these findings, with IFNG being the putative regulator with the highest significance. In BrM, this upstream regulator analysis further demonstrated the importance of mTOR signaling but also showed that *TCF7L2* and *SH3TC2* may be important players in BrM development. TCF7L2 variants have been found to be associated with breast cancer incidence (93, 94). Additionally, this gene is a positive regulator of Wnt signaling, regulates the MYC oncogene, represses the cell cycle inhibitors CDKN2C/CDKN2D, and is a transcriptional driver of various oncogenes, contributing to the progression of colon cancer and other cancer types (95).

We performed survival analyses examining the prognostic potential of IGS in the context of standard clinicopathological features. Signatures representing levels of T cells, B cells, NK cells, and DC cells in the primary tumors were associated with improved survival. The collective association of these IGS with favorable survival is likely indicative of the high degree of expression correlation structure (and thus co-infiltration levels of the associated immune cell types) observed in this cohort (**Supplementary Figure S5**). Moreover, the favorable association between T cell, B cell, and DC IGS and survival may indicate that patients with higher anti-tumor immune infiltrate in their primary TNBC may have a higher propensity to develop long-lasting immunological memory that functions to stave off metastatic spread. Similarly, levels of signatures reflective of gamma-delta T cells and ICB responsiveness [*e*.*g*., Vincent_IPRES_Responder signature (96)] in BrM were associated with improved survival from the time of BrM diagnosis, indicating that elevated immune involvement in the brain TME may be beneficial to patient survival. Conversely, the aforementioned fibroblast wound healing signature [Chang_Serum_Response_Up (64)] was associated with poor survival in BrM, indicative of a deleterious quality of this signature and the underlying biology that it represents.

While this study represents the largest series focused on TNBC BrM to date, to our knowledge, it is mainly limited by low power, particularly regarding matched pairs (with only *n* = 6 matched WES and *n* = 9 matched RNA-seq pairs). An additional limitation is the inability to corroborate adaptive immune receptor repertoire inference with amplicon sequencing, which was precluded due to inadequate specimen nucleic acid abundance. Future work will expand these, and additional analyses to additional TNBC samples, as well as to other BC subtypes in the LCCC1419 biobank, including HER2+ and ER/PR+ BCBrM, to enable a comparison of BrM across the spectrum of BC. Utilization of *in vivo* murine models for testing the relevance of these findings, including the assessment of vaccine strategies and ICB as potential therapeutic approaches for TNBC BrM, is warranted.

In summary, we report the genomic characterization of BrM compared to primary tumors from TNBC patients, including some matched pairs, with a focus on the immune landscape. Utilizing both WES and RNA-seq analytical pipelines, we demonstrated that BrM exhibited increased TMB and SNV mutational load, reduced immune gene signature expression and TCR receptor abundance/diversity metrics, and increased expression of a wound healing signature. A prediction of elevated levels of CTA- and ERV-specific neoantigen peptides was confirmed in both anatomical locations, supporting the continued development of vaccine and immune checkpoint inhibition approaches in TNBC. IGS, including T cell-related immune signatures in primary and BrM TNBC, correlated with improved survival in this patient cohort. We expect that these results and the data reported herein will be valuable in understanding TNBC BrM biology going forward and provide further rationale for the application of immunotherapeutic approaches in this disease.

## Data Availability Statement

The datasets presented in this study can be found in online repositories. The name of the repository and accession number can be found below:

https://www.ncbi.nlm.nih.gov/projects/gap/cgi-bin/study.cgi?study_id=phs002457.v1.p1.

## Ethics Statement

The studies involving human participants were reviewed and approved by the University of North Carolina at Chapel Hill Institutional Review Board. The patients/participants provided their written informed consent to participate in this study.

## Author Contributions

Conception: CA, BV, MM, and LCa. Design: ER, AVS, MS, MM, BV, SC, CA, and LiC. Acquisition/analysis: MS, LuC, LiC, AG, AW, ER, SV, SC, MW, JP, MM, AVS, BV, CA, and AH. Interpretation: ER, AVS, MS, SC, JP, MM, MW, CA, and BV. Manuscript preparation/editing: all authors. All authors contributed to the article and approved the submitted version.

## Funding

This study received funding from Susan G. Komen Career Catalyst Award (BV), V Foundation for Cancer Research Translational Grant (BV and CA), AACR grant (CA), and Translating Duke Health (CA).

## Conflict of Interest

JP is an inventor on patent applications for the Breast PAM50 assay. BV and JP report equity and consulting fees for GeneCentric Therapeutics. CA receives research funding from PUMA, Lilly, Merck, Seattle Genetics, Nektar, Tesaro, G1-Therapeutics, ZION, Novartis, and Pfizer; compensated consultant role: Genentech (1/2019-), Eisai (1/2019-), IPSEN (2/2019-), Seattle Genetics (11/15/2019-11/15/2020); Astra Zeneca (3/2020-6/2020), Novartis (5/2020-5/2022), Immunomedics (10/1/2020-9/22/2021), Elucida (9/2020); and royalties from UpToDate and Jones and Bartlett. LC has received institutional research funding from Syndax, Novartis, NanoString Technologies, AbbVie, Seattle Genetics, and Veracyte. An immediate family member has a royalty-sharing agreement and investorship interest in licensed IP to startup company Falcon Therapeutics that is designing neural stem-cell-based therapy for glioblastoma multiforme. She has uncompensated relationships with Sanofi, Novartis, G1 Therapeutics, Genentech/Roche, GlaxoSmithKline, AstraZeneca/Daiichi Sankyo, Aptitude Health, Exact Sciences and Eisai.

The remaining authors declare that the research was conducted in the absence of any commercial or financial relationships that could be construed as a potential conflict of interest.

## Publisher’s Note

All claims expressed in this article are solely those of the authors and do not necessarily represent those of their affiliated organizations, or those of the publisher, the editors and the reviewers. Any product that may be evaluated in this article, or claim that may be made by its manufacturer, is not guaranteed or endorsed by the publisher.

## Supplementary Material

Supplemental Figure 1 Samples included in immunohistochemical and sequencing (RNA and DNA) analyses. Primary breast tumor, BrM, and normal blood specimens from a total of 25 patients with BrM from TNBC were included in the analyses. Matched blood was analyzed by DNA sequencing as a comparison for tumor WES for identification of somatic variants. Final sample numbers by tissue and analysis type are provided.

Supplemental Table 1 Clinicopathological characteristics and specimen availability by patient in the LCCC1419 study cohort. Grayed boxes indicate tumors that switched subtypes between the primary tumor and the BrM based on IHC (n=2 patients). “Y” indicates a specimen was analyzed by the indicated method.

Supplemental Figure 2 PAM50 intrinsic molecular subtype analysis of RNA-Seq expression data. **(A)** Heatmap displaying that the majority of samples in 1419 were of the basal subtype. Subtype analysis was performed according to methods described in Picornell et al27. Red circles highlight samples from the two patients where IHC indicated subtype switching (see **Table 1**). The R package heatmaply97 was used for heatmap visualization with hierarchical clustering based on average linkage. **(B)** Stacked barplot displaying percentage of primary and BrM tumors belonging to respective molecular subtypes.

Supplemental Figure 3 Intrasample variant sharing is minimal in primary and BrM TNBC. Circos plot showing total number of variants and proportion of variants shared between primary TNBC (yellow; n=13) and BrM (purple; n=17).

Supplemental Figure 4 TMB is associated with survival in the LCCC1419 TNBC cohort. Multivariable survival analysis including age at diagnosis, race, stage, and TMB of primary tumor as covariates relative to time to event, which for this analysis was the time from diagnosis of the primary tumor to death. Patients with unknown race or stage were excluded from analysis (n=11 patients included). Additionally for this analysis stage I and II were binned, and stage III and IV were binned.

Supplemental Figure 5 Somatic copy number alteration assessment. SCNA in **(A)** primary TNBC (n=12) and **(B)** BrM (n=16) as determined by Sequenza/GISTIC 2.0 analysis. Significant amplicons (Amp) or deleted (Del) regions are annotated (q<0.25, green line). 11p13 amplification and 13q11 deletion, which are shared genomic features of primary and BrM TNBC, are highlighted by colored boxes. Potential oncogenes and tumor suppressors are annotated on the plots, and stars indicate genomic regions where copy number alterations are known to contribute to breast cancer/aggressive basal breast cancer.

Supplemental Figure 6 Ploidy, tumor purity, and subclonal heterogeneity assessment of LCCC1419. **(A)** Ploidy and **(B)** tumor purity were assessed using Sequenza, where no significant differences were observed between primary TNBC (n=12) and BrM (n=16); matched pairs shown on right of each panel (n=6). **(C)** Distribution of subclone number per tumor. **(D)** Cellular prevalence and mutational load of subclones in matched pairs (n=6).

Supplemental Figure 7 Correlation matrix of expression of immune gene signatures (IGS) used in this study. **(A)** IGS (n=32; refer to Supplemental Data 1 for genes comprising respective signatures) were quantified from RNA-Seq expression data from primary TNBC (n=15) and BrM (n=19), and analyzed by Spearman correlation analysis. **(B)** IGS correlation matrix applied to the same dataset from **(A)** using a compendium of IGS (64 signatures; refer to Supplemental Data 1 for genes comprising respective signatures) curated by our group. Due to the high degree of correlation of some of the subsets of IGS, we culled this list to the 32 signatures shown in **(A)** so as to reduce signature redundancy while maintaining breadth of represented immune cell types/features. Color denotes Spearman rho, and X indicates a non-significant relationship (p>0.05). The R package Ggcorrplot98 was used to generate correlation plot.

Supplemental Figure 8 Immune cell type deconvolution of RNA-Seq expression data. RNA-Seq expression data from primary TNBC (n=15) and BrM (n=19) was input into CIBERSORTx (33) (https://cibersortx.stanford.edu/) to determine relative contributions of 22 immune cell subtypes (LM22) to tumor composition. Wilcoxon rank-sum test was performed on Z-transformed cell fraction values to determine statistical significance. Significance codes: *q<0.05; **q<0.01.

Supplemental Figure 9 Analysis of additional immune modules further demonstrate immune cell deficit in BrM relative to primary TNBC. **(A)** Expression of the 20-gene Immunologic Constant of Rejection (ICR) signature was significantly reduced in BrM (n=19) relative to primary TNBC (n=15). **(B)** Blood transcriptional module repertoires reported by Rinchai et al (DOI: 10.1093/bioinformatics/btab121) were applied to the LCCC1419 RNAseq dataset using the Bioconductor R package BloodGen3Module Groupcomparison function. Significant immune modules are annotated on the plot. This analysis corroborates data showing a reduction of B and T cells in the BrM relative to primary TNBC (q<0.1).

Supplemental Figure 10 Multivariable survival analysis of clinicopathological variables. Clinicopathological variables (age at primary tumor diagnosis, race, and stage) were analyzed by multivariable CoxPH. Category labels indicate time metric to event that was applied. Patients with unknown race (including one Asian patient) or stage were excluded from analysis (n=21 patients included). Additionally for this analysis stage I and II were binned, and stage III and IV were binned. Significance codes: *q<0.05; **q<0.01; ns, not significant.

Supplemental Figure 11 Multivariable CoxPH analysis of IGS in primary and BrM TNBC. Each of 32 IGS (refer to **Supplemental Figure 7**) was included as a covariate along with clinicopathological variables (age at diagnosis, race, stage), which yielded odds ratios (OR; hazard ratios) and p values. These p values were then FDR-adjusted. This analysis was performed using three different time metrics to death, as indicated in **(A)** primary TNBC and **(B)** BrM analyses. Only IGS that had unadjusted p values of < 0.1 are shown. Note that none of the IGS achieved significant association with survival after false discovery correction, which is likely due to low sample number.

## References

[1] Brosnan EM Anders CK. Understanding Patterns of Brain Metastasis in Breast Cancer and Designing Rational Therapeutic Strategies. Ann Transl Med (2018) 6(9):163. doi: 10.21037/atm.2018.04.35 29911111 PMC5985267

[2] Niwinska A Murawska M Pogoda K. Breast Cancer Brain Metastases: Differences in Survival Depending on Biological Subtype, RPA RTOG Prognostic Class and Systemic Treatment After Whole-Brain Radiotherapy (WBRT). Ann Oncol (2010) 21(5):942–8. doi: 10.1093/annonc/mdp407 19840953

[3] Diéras V Weaver R Tolaney SM Bardia A Punie K Brufsky A. Abstract PD13-07: Subgroup Analysis of Patients With Brain Metastases From the Phase 3 ASCENT Study of Sacituzumab Govitecan Versus Chemotherapy in Metastatic Triple-Negative Breast Cancer. Cancer Res (2021) 81(4 Supplement):PD13–07-PD13-07. doi: 10.1158/1538-7445.Sabcs20-pd13-07

[4] Fischer GM Jalali A Kircher DA Lee WC McQuade JL Haydu LE. Molecular Profiling Reveals Unique Immune and Metabolic Features of Melanoma Brain Metastases. Cancer Discov (2019) 9(5):628–45. doi: 10.1158/2159-8290.CD-18-1489 PMC6497554 30787016

[5] Shih DJH Nayyar N Bihun I Dagogo-Jack I Gill CM Aquilanti E. Genomic Characterization of Human Brain Metastases Identifies Drivers of Metastatic Lung Adenocarcinoma. Nat Genet (2020) 52(4):371–7. doi: 10.1038/s41588-020-0592-7 PMC7136154 32203465

[6] Salhia B Kiefer J Ross JT Metapally R Martinez RA Johnson KN. Integrated Genomic and Epigenomic Analysis of Breast Cancer Brain Metastasis. PloS One (2014) 9(1):e85448. doi: 10.1371/journal.pone.0085448 24489661 PMC3906004

[7] Priedigkeit N Hartmaier RJ Chen Y Vareslija D Basudan A Watters RJ. Intrinsic Subtype Switching and Acquired ERBB2/HER2 Amplifications and Mutations in Breast Cancer Brain Metastases. JAMA Oncol (2017) 3(5):666–71. doi: 10.1001/jamaoncol.2016.5630 PMC5508875 27926948

[8] Van Swearingen AED Siegel MB Deal AM Sambade MJ Hoyle A Hayes DN. LCCC 1025: A Phase II Study of Everolimus, Trastuzumab, and Vinorelbine to Treat Progressive HER2-Positive Breast Cancer Brain Metastases. Breast Cancer Res Treat (2018) 171(3):637–48. doi: 10.1007/s10549-018-4852-5 PMC6779035 29938395

[9] Brastianos PK Carter SL Santagata S Cahill DP Taylor-Weiner A Jones RT. Genomic Characterization of Brain Metastases Reveals Branched Evolution and Potential Therapeutic Targets. Cancer Discov (2015) 5(11):1164–77. doi: 10.1158/2159-8290.CD-15-0369 PMC4916970 26410082

[10] Li H Handsaker B Wysoker A Fennell T Ruan J Homer N. The Sequence Alignment/Map Format and SAMtools. Bioinformatics (2009) 25(16):2078–9. doi: 10.1093/bioinformatics/btp352 PMC2723002 19505943

[11] Malin D Strekalova E Petrovic V Deal AM Al Ahmad A Adamo B. alphaB-Crystallin: A Novel Regulator of Breast Cancer Metastasis to the Brain. Clin Cancer Res (2014) 20(1):56–67. doi: 10.1158/1078-0432.CCR-13-1255 24132917 PMC3973485

[12] Ippen FM Alvarez-Breckenridge CA Kuter BM Fink AL Bihun IV Lastrapes M. The Dual PI3K/mTOR Pathway Inhibitor GDC-0084 Achieves Antitumor Activity in PIK3CA-Mutant Breast Cancer Brain Metastases. Clin Cancer Res (2019) 25(11):3374–83. doi: 10.1158/1078-0432.CCR-18-3049 PMC6685218 30796030

[13] Ippen FM Grosch JK Subramanian M Kuter BM Liederer BM Plise EG. Targeting the PI3K/Akt/mTOR Pathway With the Pan-Akt Inhibitor GDC-0068 in PIK3CA-Mutant Breast Cancer Brain Metastases. Neuro Oncol (2019) 21(11):1401–11. doi: 10.1093/neuonc/noz105 PMC6827829 31173106

[14] Kardos J Chai S Mose LE Selitsky SR Krishnan B Saito R. Claudin-Low Bladder Tumors Are Immune Infiltrated and Actively Immune Suppressed. JCI Insight (2016) 1(3):e85902. doi: 10.1172/jci.insight.85902 27699256 PMC5033914

[15] Turajlic S Litchfield K Xu H Rosenthal R McGranahan N Reading JL. Insertion-And-Deletion-Derived Tumour-Specific Neoantigens and the Immunogenic Phenotype: A Pan-Cancer Analysis. Lancet Oncol (2017) 18(8):1009–21. doi: 10.1016/S1470-2045(17)30516-8 28694034

[16] Zhang J Mardis ER Maher CA. INTEGRATE-Neo: A Pipeline for Personalized Gene Fusion Neoantigen Discovery. Bioinformatics (2017) 33(4):555–7. doi: 10.1093/bioinformatics/btw674 PMC5408800 27797777

[17] Jayasinghe RG Cao S Gao Q Wendl MC Vo NS Reynolds SM. Systematic Analysis of Splice-Site-Creating Mutations in Cancer. Cell Rep (2018) 23(1):270–281e273. doi: 10.1016/j.celrep.2018.03.052 29617666 PMC6055527

[18] Lansford JL Dharmasiri U Chai S Hunsucker SA Bortone DS Keating JE. Computational Modeling and Confirmation of Leukemia-Associated Minor Histocompatibility Antigens. Blood Adv (2018) 2(16):2052–62. doi: 10.1182/bloodadvances.2018022475 PMC6113610 30115642

[19] Saito R Smith CC Utsumi T Bixby LM Kardos J Wobker SE. Molecular Subtype-Specific Immunocompetent Models of High-Grade Urothelial Carcinoma Reveal Differential Neoantigen Expression and Response to Immunotherapy. Cancer Res (2018) 78(14):3954–68. doi: 10.1158/0008-5472.CAN-18-0173 PMC6157276 29784854

[20] Smith CC Beckermann KE Bortone DS De Cubas AA Bixby LM Lee SJ. Endogenous Retroviral Signatures Predict Immunotherapy Response in Clear Cell Renal Cell Carcinoma. J Clin Invest (2018) 128(11):4804–20. doi: 10.1172/jci121476 PMC6205406 30137025

[21] Thorsson V Gibbs DL Brown SD Wolf D Bortone DS Ou Yang TH. The Immune Landscape of Cancer. Immunity (2018) 48(4):812–830e814. doi: 10.1016/j.immuni.2018.03.023 29628290 PMC5982584

[22] Smith CC Chai S Washington AR Lee SJ Landoni E Field K. Machine-Learning Prediction of Tumor Antigen Immunogenicity in the Selection of Therapeutic Epitopes. Cancer Immunol Res (2019) 7(10):1591–604. doi: 10.1158/2326-6066.CIR-19-0155 PMC6774822 31515258

[23] Buchkovich ML Brown CC Robasky K Chai S Westfall S Vincent BG. HLAProfiler Utilizes K-Mer Profiles to Improve HLA Calling Accuracy for Rare and Common Alleles in RNA-Seq Data. Genome Med (2017) 9(1):86. doi: 10.1186/s13073-017-0473-6 28954626 PMC5618726

[24] Jurtz V Paul S Andreatta M Marcatili P Peters B Nielsen M. NetMHCpan-4.0: Improved Peptide-MHC Class I Interaction Predictions Integrating Eluted Ligand and Peptide Binding Affinity Data. J Immunol (2017) 199(9):3360–8. doi: 10.4049/jimmunol.1700893 PMC5679736 28978689

[25] Hunsucker SA McGary CS Vincent BG Enyenihi AA Waugh JP McKinnon KP. Peptide/MHC Tetramer-Based Sorting of CD8(+) T Cells to a Leukemia Antigen Yields Clonotypes Drawn Nonspecifically From an Underlying Restricted Repertoire. Cancer Immunol Res (2015) 3(3):228–35. doi: 10.1158/2326-6066.CIR-14-0001 PMC4351150 25576336

[26] Zhao W He X Hoadley KA Parker JS Hayes DN Perou CM. Comparison of RNA-Seq by Poly (A) Capture, Ribosomal RNA Depletion, and DNA Microarray for Expression Profiling. BMC Genomics (2014) 15(1):419. doi: 10.1186/1471-2164-15-419 24888378 PMC4070569

[27] Picornell AC Echavarria I Alvarez E Lopez-Tarruella S Jerez Y Hoadley K. Breast Cancer PAM50 Signature: Correlation and Concordance Between RNA-Seq and Digital Multiplexed Gene Expression Technologies in a Triple Negative Breast Cancer Series. BMC Genomics (2019) 20(1):452. doi: 10.1186/s12864-019-5849-0 31159741 PMC6547580

[28] Galili T O'Callaghan A Sidi J Sievert C. Heatmaply: An R Package for Creating Interactive Cluster Heatmaps for Online Publishing. Bioinformatics (2018) 34(9):1600–2. doi: 10.1093/bioinformatics/btx657 PMC5925766 29069305

[29] Bortone D. "Binfotron: Binfotron Bioinformatics Analysis Tools Suite" In (R package version 0.3-17 ed.). (2020).

[30] Love MI Huber W Anders S. Moderated Estimation of Fold Change and Dispersion for RNA-Seq Data With Deseq2. Genome Biol (2014) 15(12):550. doi: 10.1186/s13059-014-0550-8 25516281 PMC4302049

[31] Hänzelmann S Castelo R Guinney J. GSVA: Gene Set Variation Analysis for Microarray and RNA-Seq Data. BMC Bioinf (2013) 14:7. doi: 10.1186/1471-2105-14-7 PMC3618321 23323831

[32] Morgan M Falcon S Gentleman R. "GSEABase: Gene Set Enrichmentdata Structures and Methods" In (R package version 1.48.0 ed.). (2019).

[33] Newman AM Liu CL Green MR Gentles AJ Feng W Xu Y. Robust Enumeration of Cell Subsets From Tissue Expression Profiles. Nat Methods (2015) 12(5):453–7. doi: 10.1038/nmeth.3337 PMC4739640 25822800

[34] Bolotin DA Poslavsky S Mitrophanov I Shugay M Mamedov IZ Putintseva EV. MiXCR: Software for Comprehensive Adaptive Immunity Profiling. Nat Methods (2015) 12(5):380–1. doi: 10.1038/nmeth.3364 25924071

[35] Mose LE Selitsky SR Bixby LM Marron DL Iglesia MD Serody JS. Assembly-Based Inference of B-Cell Receptor Repertoires From Short Read RNA Sequencing Data With V'DJer. Bioinformatics (2016) 32(24):3729–34. doi: 10.1093/bioinformatics/btw526 PMC5167060 27559159

[36] Patro R Duggal G Love MI Irizarry RA Kingsford C. Salmon Provides Fast and Bias-Aware Quantification of Transcript Expression. Nat Methods (2017) 14(4):417–9. doi: 10.1038/nmeth.4197 PMC5600148 28263959

[37] Bortone DS Woodcock MG Parker JS Vincent BG. Improved T-Cell Receptor Diversity Estimates Associate With Survival and Response to Anti-PD-1 Therapy. Cancer Immunol Res (2021) 9(1):103–12. doi: 10.1158/2326-6066.CIR-20-0398 33177107

[38] Therneau TM. "A Package for Survival Analysis'' in R". version 2.38 ed. (2015).

[39] Max Gordon TL. "Forestplot: Advanced Forest Plot Using 'Grid' Graphics" In version 1.7.2 ed. (2017).

[40] Rajasagi M Shukla SA Fritsch EF Keskin DB DeLuca D Carmona E. Systematic Identification of Personal Tumor-Specific Neoantigens in Chronic Lymphocytic Leukemia. Blood (2014) 124(3):453–62. doi: 10.1182/blood-2014-04-567933 PMC4102716 24891321

[41] Litchfield K Reading JL Puttick C Thakkar K Abbosh C Bentham R. Meta-Analysis of Tumor- and T Cell-Intrinsic Mechanisms of Sensitization to Checkpoint Inhibition. Cell (2021) 184(3):596–614e514. doi: 10.1016/j.cell.2021.01.002 33508232 PMC7933824

[42] Tate JG Bamford S Jubb HC Sondka Z Beare DM Bindal N. COSMIC: The Catalogue Of Somatic Mutations In Cancer. Nucleic Acids Res (2019) 47(D1):D941–7. doi: 10.1093/nar/gky1015 PMC6323903 30371878

[43] Cancer Genome Atlas N. Comprehensive Molecular Portraits of Human Breast Tumours. Nature (2012) 490(7418):61–70. doi: 10.1038/nature11412 23000897 PMC3465532

[44] Mermel CH Schumacher SE Hill B Meyerson ML Beroukhim R Getz G. GISTIC2.0 Facilitates Sensitive and Confident Localization of the Targets of Focal Somatic Copy-Number Alteration in Human Cancers. Genome Biol (2011) 12(4):R41. doi: 10.1186/gb-2011-12-4-r41 21527027 PMC3218867

[45] Favero F Joshi T Marquard AM Birkbak NJ Krzystanek M Li Q. Sequenza: Allele-Specific Copy Number and Mutation Profiles From Tumor Sequencing Data. Ann Oncol (2015) 26(1):64–70. doi: 10.1093/annonc/mdu479 25319062 PMC4269342

[46] Klingbeil P Natrajan R Everitt G Vatcheva R Marchio C Palacios J. CD44 is Overexpressed in Basal-Like Breast Cancers But is Not a Driver of 11p13 Amplification. Breast Cancer Res Treat (2010) 120(1):95–109. doi: 10.1007/s10549-009-0380-7 19350388

[47] Artibani M Sims AH Slight J Aitken S Thornburn A Muir M. WT1 Expression in Breast Cancer Disrupts the Epithelial/Mesenchymal Balance of Tumour Cells and Correlates With the Metabolic Response to Docetaxel. Sci Rep (2017) 7:45255. doi: 10.1038/srep45255 28345629 PMC5366898

[48] Rouault A Banneau G Macgrogan G Jones N Elarouci N Barouk-Simonet E. Deletion of Chromosomes 13q and 14q Is a Common Feature of Tumors With BRCA2 Mutations. PloS One (2012) 7(12):e52079. doi: 10.1371/journal.pone.0052079 23284877 PMC3528765

[49] Farabegoli F Ceccarelli C Santini D Trere D Baldini N Taffurelli M. Chromosome 1 Aneusomy With 1p36 Under-Representation Is Related to Histologic Grade, DNA Aneuploidy, High C-Erb B-2 and Loss of Bcl-2 Expression in Ductal Breast Carcinoma. Int J Cancer (1996) 69(5):381–5. doi: 10.1002/(SICI)1097-0215(19961021)69:5<381::AID-IJC5>3.0.CO;2-1 8900371

[50] Shivapurkar N Sood S Wistuba II Virmani AK Maitra A Milchgrub S. Multiple Regions of Chromosome 4 Demonstrating Allelic Losses in Breast Carcinomas. Cancer Res (1999) 59(15):3576–80. 10446964

[51] Martinez A Walker RA Shaw JA Dearing SJ Maher ER Latif F. Chromosome 3p Allele Loss in Early Invasive Breast Cancer: Detailed Mapping and Association With Clinicopathological Features. Mol Pathol (2001) 54(5):300–6. doi: 10.1136/mp.54.5.300 PMC1187086 11577171

[52] Oesterreich S Allredl DC Mohsin SK Zhang Q Wong H Lee AV. High Rates of Loss of Heterozygosity on Chromosome 19p13 in Human Breast Cancer. Br J Cancer (2001) 84(4):493–8. doi: 10.1054/bjoc.2000.1606 PMC2363776 11207044

[53] Yang TL Su YR Huang CS Yu JC Lo YL Wu PE. High-Resolution 19p13.2-13.3 Allelotyping of Breast Carcinomas Demonstrates Frequent Loss of Heterozygosity. Genes Chromosomes Cancer (2004) 41(3):250–6. doi: 10.1002/gcc.20080 15334548

[54] Letessier A Sircoulomb F Ginestier C Cervera N Monville F Gelsi-Boyer V. Frequency, Prognostic Impact, and Subtype Association of 8p12, 8q24, 11q13, 12p13, 17q12, and 20q13 Amplifications in Breast Cancers. BMC Cancer (2006) 6:245. doi: 10.1186/1471-2407-6-245 17040570 PMC1626089

[55] Melchor L Saucedo-Cuevas LP Munoz-Repeto I Rodriguez-Pinilla SM Honrado E Campoverde A. Comprehensive Characterization of the DNA Amplification at 13q34 in Human Breast Cancer Reveals TFDP1 and CUL4A as Likely Candidate Target Genes. Breast Cancer Res (2009) 11(6):R86. doi: 10.1186/bcr2456 19995430 PMC2815550

[56] Yu W Kanaan Y Bae YK Gabrielson E. Chromosomal Changes in Aggressive Breast Cancers With Basal-Like Features. Cancer Genet Cytogenet (2009) 193(1):29–37. doi: 10.1016/j.cancergencyto.2009.03.017 19602461 PMC2768045

[57] Zhang J Liu X Datta A Govindarajan K Tam WL Han J. RCP is a Human Breast Cancer-Promoting Gene With Ras-Activating Function. J Clin Invest (2009) 119(8):2171–83. doi: 10.1172/JCI37622 PMC2719918 19620787

[58] Wikman H Sielaff-Frimpong B Kropidlowski J Witzel I Milde-Langosch K Sauter G. Clinical Relevance of Loss of 11p15 in Primary and Metastatic Breast Cancer: Association With Loss of PRKCDBP Expression in Brain Metastases. PloS One (2012) 7(10):e47537. doi: 10.1371/journal.pone.0047537 23118876 PMC3485301

[59] Chen YY Ge JY Zhu SY Shao ZM Yu KD. Copy Number Amplification of ENSA Promotes the Progression of Triple-Negative Breast Cancer *via* Cholesterol Biosynthesis. Nat Commun (2022) 13(1):791. doi: 10.1038/s41467-022-28452-z 35145111 PMC8831589

[60] Rieber N Bohnert R Ziehm U Jansen G. Reliability of Algorithmic Somatic Copy Number Alteration Detection From Targeted Capture Data. Bioinformatics (2017) 33(18):2791–8. doi: 10.1093/bioinformatics/btx284 PMC5870863 28472276

[61] Tanner G Westhead DR Droop A Stead LF. Benchmarking Pipelines for Subclonal Deconvolution of Bulk Tumour Sequencing Data. Nat Commun (2021) 12(1):6396. doi: 10.1038/s41467-021-26698-7 34737285 PMC8569188

[62] Talevich E Shain AH Botton T Bastian BC. CNVkit: Genome-Wide Copy Number Detection and Visualization From Targeted DNA Sequencing. PloS Comput Biol (2016) 12(4):e1004873. doi: 10.1371/journal.pcbi.1004873 27100738 PMC4839673

[63] Gillis S Roth A. PyClone-VI: Scalable Inference of Clonal Population Structures Using Whole Genome Data. BMC Bioinf (2020) 21(1):571. doi: 10.1186/s12859-020-03919-2 PMC7730797 33302872

[64] Smith CC Selitsky SR Chai S Armistead PM Vincent BG Serody JS. Alternative Tumour-Specific Antigens. Nat Rev Cancer (2019) 19(8):465–78. doi: 10.1038/s41568-019-0162-4 PMC6874891 31278396

[65] Chang HY Sneddon JB Alizadeh AA Sood R West RB Montgomery K. Gene Expression Signature of Fibroblast Serum Response Predicts Human Cancer Progression: Similarities Between Tumors and Wounds. PloS Biol (2004) 2(2):E7. doi: 10.1371/journal.pbio.0020007 14737219 PMC314300

[66] Newman AM Steen CB Liu CL Gentles AJ Chaudhuri AA Scherer F. Determining Cell Type Abundance and Expression From Bulk Tissues With Digital Cytometry. Nat Biotechnol (2019) 37(7):773–82. doi: 10.1038/s41587-019-0114-2 PMC6610714 31061481

[67] Bertucci F Finetti P Simeone I Hendrickx W Wang E Marincola FM. The Immunologic Constant of Rejection Classification Refines the Prognostic Value of Conventional Prognostic Signatures in Breast Cancer. Br J Cancer (2018) 119(11):1383–91. doi: 10.1038/s41416-018-0309-1 PMC6265245 30353048

[68] Rinchai D Roelands J Toufiq M Hendrickx W Altman MC Bedognetti D. BloodGen3Module: Blood Transcriptional Module Repertoire Analysis and Visualization Using R. Bioinformatics (2021). doi: 10.1093/bioinformatics/btab121 PMC8388021 33624743

[69] Robins HS Campregher PV Srivastava SK Wacher A Turtle CJ Kahsai O. Comprehensive Assessment of T-Cell Receptor Beta-Chain Diversity in Alphabeta T Cells. Blood (2009) 114(19):4099–107. doi: 10.1182/blood-2009-04-217604 PMC2774550 19706884

[70] Carlson CS Emerson RO Sherwood AM Desmarais C Chung MW Parsons JM. Using Synthetic Templates to Design an Unbiased Multiplex PCR Assay. Nat Commun (2013) 4:2680. doi: 10.1038/ncomms3680 24157944

[71] Gao YK Kuksis M Id Said B Chehade R Kiss A Tran W. Treatment Patterns and Outcomes of Women With Symptomatic and Asymptomatic Breast Cancer Brain Metastases: A Single-Center Retrospective Study. Oncologist (2021) 26(11):e1951-e1961. doi: 10.1002/onco.13965 34506676 PMC8571756

[72] Zimmerman BS Seidman D Cascetta KP Ru M Moshier E Tiersten A. Prognostic Factors and Survival Outcomes Among Patients With Breast Cancer and Brain Metastases at Diagnosis: A National Cancer Database Analysis. Oncology (2021) 99(5):280–91. doi: 10.1159/000512212 33652435

[73] Sammons S Van Swearingen AED Anders CK. The Promise of Immunotherapy for Breast Cancer Brain Metastases. Curr Breast Cancer Rep (2019). doi: 10.1007/s12609-019-00335-1

[74] Lehmann BD Bauer JA Chen X Sanders ME Chakravarthy AB Shyr Y. Identification of Human Triple-Negative Breast Cancer Subtypes and Preclinical Models for Selection of Targeted Therapies. J Clin Invest (2011) 121(7):2750–67. doi: 10.1172/JCI45014 PMC3127435 21633166

[75] Loizidou MA Cariolou MA Neuhausen SL Newbold RF Bashiardes E Marcou Y. Genetic Variation in Genes Interacting With BRCA1/2 and Risk of Breast Cancer in the Cypriot Population. Breast Cancer Res Treat (2010) 121(1):147–56. doi: 10.1007/s10549-009-0518-7 19714462

[76] Giuli MV Giuliani E Screpanti I Bellavia D Checquolo S. Notch Signaling Activation as a Hallmark for Triple-Negative Breast Cancer Subtype. J Oncol (2019) 2019:8707053. doi: 10.1155/2019/8707053 31379945 PMC6657611

[77] Sheffer M Bacolod MD Zuk O Giardina SF Pincas H Barany F. Association of Survival and Disease Progression With Chromosomal Instability: A Genomic Exploration of Colorectal Cancer. Proc Natl Acad Sci USA (2009) 106(17):7131–6. doi: 10.1073/pnas.0902232106 PMC2678450 19359472

[78] Titus AJ Way GP Johnson KC Christensen BC. Deconvolution of DNA Methylation Identifies Differentially Methylated Gene Regions on 1p36 Across Breast Cancer Subtypes. Sci Rep (2017) 7(1):11594. doi: 10.1038/s41598-017-10199-z 28912426 PMC5599639

[79] Gelsi-Boyer V Orsetti B Cervera N Finetti P Sircoulomb F Rouge C. Comprehensive Profiling of 8p11-12 Amplification in Breast Cancer. Mol Cancer Res (2005) 3(12):655–67. doi: 10.1158/1541-7786.MCR-05-0128 16380503

[80] Mesquita B Lopes P Rodrigues A Pereira D Afonso M Leal C. Frequent Copy Number Gains at 1q21 and 1q32 are Associated With Overexpression of the ETS Transcription Factors ETV3 and ELF3 in Breast Cancer Irrespective of Molecular Subtypes. Breast Cancer Res Treat (2013) 138(1):37–45. doi: 10.1007/s10549-013-2408-2 23329352

[81] Tang X Tang J Liu X Zeng L Cheng C Luo Y. Downregulation of miR-129-2 by Promoter Hypermethylation Regulates Breast Cancer Cell Proliferation and Apoptosis. Oncol Rep (2016) 35(5):2963–9. doi: 10.3892/or.2016.4647 26935022

[82] Martorana F Motta G Pavone G Motta L Stella S Vitale SR. AKT Inhibitors: New Weapons in the Fight Against Breast Cancer? Front Pharmacol (2021) 12:662232. doi: 10.3389/fphar.2021.662232 33995085 PMC8118639

[83] Pandey V Wu ZS Zhang M Li R Zhang J Zhu T. Trefoil Factor 3 Promotes Metastatic Seeding and Predicts Poor Survival Outcome of Patients With Mammary Carcinoma. Breast Cancer Res (2014) 16(5):429. doi: 10.1186/s13058-014-0429-3 25266665 PMC4303111

[84] Al-Salam S Sudhadevi M Awwad A Al Bashir M. Trefoil Factors Peptide-3 is Associated With Residual Invasive Breast Carcinoma Following Neoadjuvant Chemotherapy. BMC Cancer (2019) 19(1):135. doi: 10.1186/s12885-019-5316-y 30744593 PMC6371459

[85] Routh ED Pullikuth AK Jin G Chifman J Chou JW D'Agostino RB Jr.. Transcriptomic Features of T Cell-Barren Tumors Are Conserved Across Diverse Tumor Types. Front Immunol (2020) 11:57. doi: 10.3389/fimmu.2020.00057 32117236 PMC7031496

[86] Mahmoud AM. Cancer Testis Antigens as Immunogenic and Oncogenic Targets in Breast Cancer. Immunotherapy (2018) 10(9):769–78. doi: 10.2217/imt-2017-0179 PMC6462849 29926750

[87] Panda A de Cubas AA Stein M Riedlinger G Kra J Mayer T. Endogenous Retrovirus Expression Is Associated With Response to Immune Checkpoint Blockade in Clear Cell Renal Cell Carcinoma. JCI Insight (2018) 3(16). doi: 10.1172/jci.insight.121522 PMC6141170 30135306

[88] Zhang SM Cai WL Liu X Thakral D Luo J Chan LH. KDM5B Promotes Immune Evasion by Recruiting SETDB1 to Silence Retroelements. Nature (2021) 598(7882):682–7. doi: 10.1038/s41586-021-03994-2 PMC8555464 34671158

[89] Louveau A Harris TH Kipnis J. Revisiting the Mechanisms of CNS Immune Privilege. Trends Immunol (2015) 36(10):569–77. doi: 10.1016/j.it.2015.08.006 PMC4593064 26431936

[90] Xiao L Zhou J Liu H Zhou Y Chen W Cui W. ). RNA Sequence Profiling Reveals Unique Immune and Metabolic Features of Breast Cancer Brain Metastases. Front Oncol (2021) 11:679262. doi: 10.3389/fonc.2021.679262 34513670 PMC8427193

[91] Dvorak HF. Tumors: Wounds That do Not Heal-Redux. Cancer Immunol Res (2015) 3(1):1–11. doi: 10.1158/2326-6066.CIR-14-0209 25568067 PMC4288010

[92] Kidman J Principe N Watson M Lassmann T Holt RA Nowak AK. Characteristics of TCR Repertoire Associated With Successful Immune Checkpoint Therapy Responses. Front Immunol (2020) 11:587014. doi: 10.3389/fimmu.2020.587014 33163002 PMC7591700

[93] Burwinkel B Shanmugam KS Hemminki K Meindl A Schmutzler RK Sutter C. Transcription Factor 7-Like 2 (TCF7L2) Variant Is Associated With Familial Breast Cancer Risk: A Case-Control Study. BMC Cancer (2006) 6:268. doi: 10.1186/1471-2407-6-268 17109766 PMC1665524

[94] Min W Liu X Lu Y Gong Z Wang M Lin S. Association of Transcription Factor 7-Like 2 Gene Polymorphisms With Breast Cancer Risk in Northwest Chinese Women. Oncotarget (2016) 7(47):77175–82. doi: 10.18632/oncotarget.12591 PMC5363578 27738320

[95] Wenzel J Rose K Haghighi EB Lamprecht C Rauen G Freihen V. Loss of the Nuclear Wnt Pathway Effector TCF7L2 Promotes Migration and Invasion of Human Colorectal Cancer Cells. Oncogene (2020) 39(19):3893–909. doi: 10.1038/s41388-020-1259-7 PMC7203011 32203164

[96] Hugo W Zaretsky JM Sun L Song C Moreno BH Hu-Lieskovan S. Genomic and Transcriptomic Features of Response to Anti-PD-1 Therapy in Metastatic Melanoma. Cell (2017) 168(3):542. doi: 10.1016/j.cell.2017.01.010 28129544

[97] Galili T O’Callaghan A Sidi J Sievert C. ). Heatmaply: An R Package for Creating Interactive Cluster Heatmaps for Online Publishing. Bioinformatics (2017) 34(9):1600–2. doi: 10.1093/bioinformatics/btx657 PMC5925766 29069305

[98] Kassambara A. "Ggcorrplot: Visualization of a Correlation Matrix Using 'Ggplot2'. (R package version 0.1.3. ed.)". (2019).

