# Supplementary material for: Comprehensive Analysis of the Immunogenomics of Triple-Negative Breast Cancer Brain Metastases From LCCC1419
Source: Front Oncol. 2022 Jul 27;12:818693. doi: 10.3389/fonc.2022.818693 (PMC9387304; doi:10.3389/fonc.2022.818693)

Supplemental Figure 1

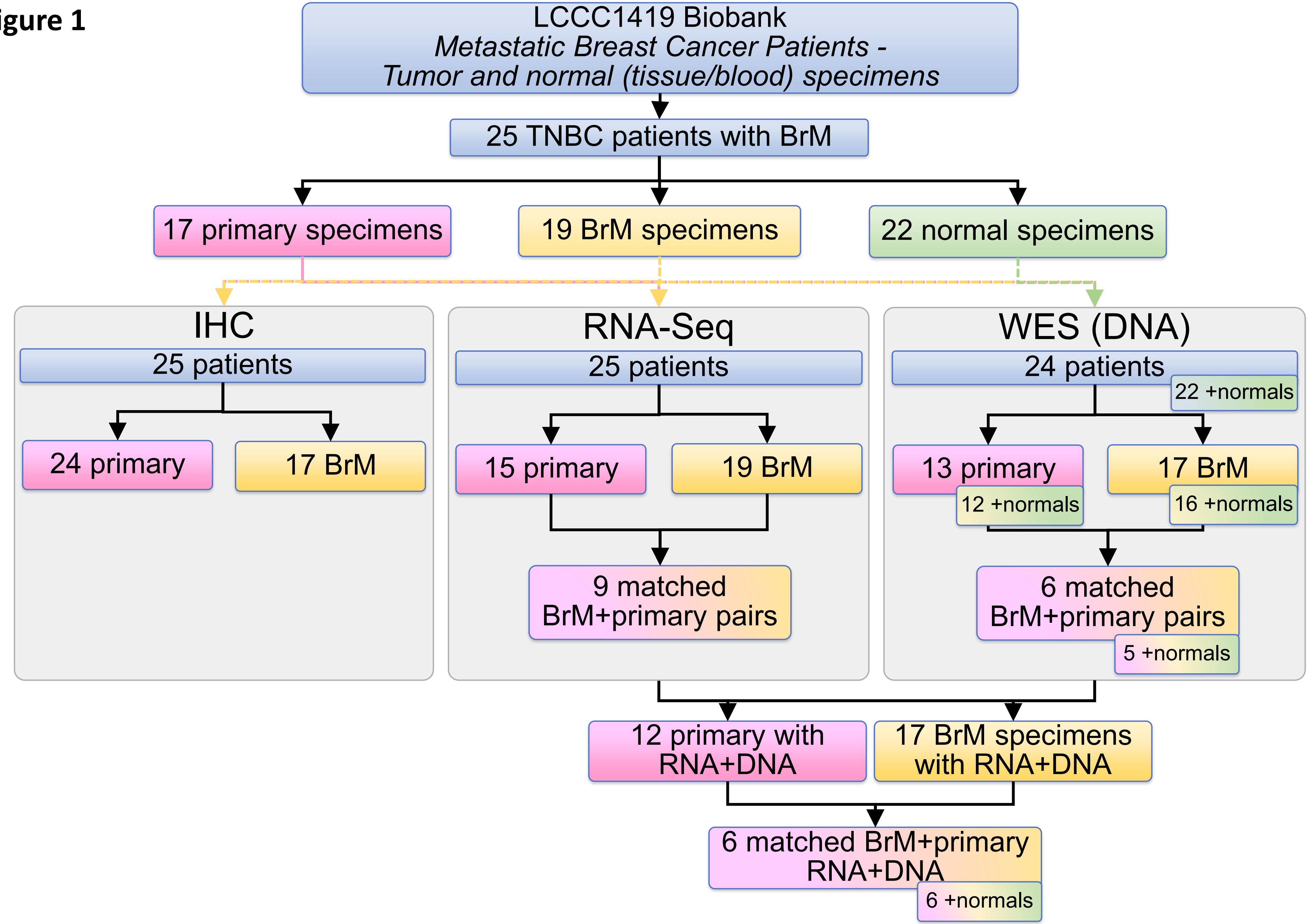

Supplemental Table 1

Patient ID	Age at Dx	Stage at Dx	Race	Sex	IHC subtype		RNA-Seq		DNA WES		
					Primary (n=24)	BrM (n=17)	Primary (n=15)	BrM (n=19)	Primary (n=13)	BrM (n=17)	Normal (blood) (n=22)
**L-01-054**	57.7	IIIB	Caucasian	F	Basal/TN	Basal/TN	Y	Y	Y	Y	Y
**L-01-062**	66	IIB	Caucasian	F	Basal/TN		Y		Y		Y
**L-01-070/L-01-071**	37.4	IIIC	Caucasian	F	Basal/TN	Not determined		Y		Y	Y
**L-01-081**	61.1	IV	African American	F	Basal/TN		Y		Y		
**L-01-082**	29.5	IIA	Caucasian	F	Basal/TN	Basal/TN		Y		Y	
**L-01-083**	47.3	IIA	Caucasian	F	Basal/TN		Y		Y		Y
**L-01-085**	50.7	IIA	Caucasian	F	Basal/TN	Basal/TN	Y	Y	Y	Y	Y
**L-01-097**	46.4	Unk	Caucasian	F	Unknown	Basal/TN		Y		Y	Y
**L-01-138**	49.5	I	Caucasian	F	Basal/TN	Basal/TN		Y	Y		Y
**L-01-153**	36.5	IIA	Caucasian	F	Basal/TN	Luminal A	Y	Y			
**L-01-175**	61.7	I	Caucasian	F	Luminal A	Basal/TN		Y		Y	Y
**L-02-012/L-03-011**	29	Unk	Caucasian	F	Basal/TN	Basal/TN	Y	Y	Y	Y	Y
**L-02-035/L-02-053**	30	Unk	Asian	F	Basal/TN		Y		Y		Y
**L-02-049**	55.5	IIB	African American	F	Basal/TN	Not determined		Y		Y	Y
**L-02-069**	46.2	IIA	Caucasian	F	Basal/TN	Basal/TN		Y		Y	Y
**L-02-076**	52	IIB	Caucasian	F	Basal/TN	Basal/TN	Y	Y		Y	Y
**L-02-101**	44.3	IV	African American	F	Basal/TN		Y		Y		Y
**L-02-104**	61.3	IIB	Caucasian	M	Basal/TN		Y		Y		Y
**L-02-108**	46.7	IIIC	Caucasian	F	Basal/TN	Basal/TN		Y		Y	Y
**L-02-119**	40.9	IIIA	African American	F	Basal/TN	Basal/TN	Y	Y	Y	Y	Y
**L-02-120**	44.8	IIIA	African American	F	Basal/TN	Basal/TN	Y	Y	Y	Y	Y
**L-02-130**	41.3	IIA	Asian	F	Basal/TN	Basal/TN		Y		Y	Y
**L-03-016**	47.5	IIA	Caucasian	F	Basal/TN	Basal/TN	Y	Y	Y	Y	Y
**L-03-026**	70.9	IIA	Caucasian	F	Basal/TN	Basal/TN	Y	Y		Y	Y
**L-03-052**	39.9	IIIA	African American	F	Basal/TN	Basal/TN		Y		Y	Y

Supplemental Figure 2

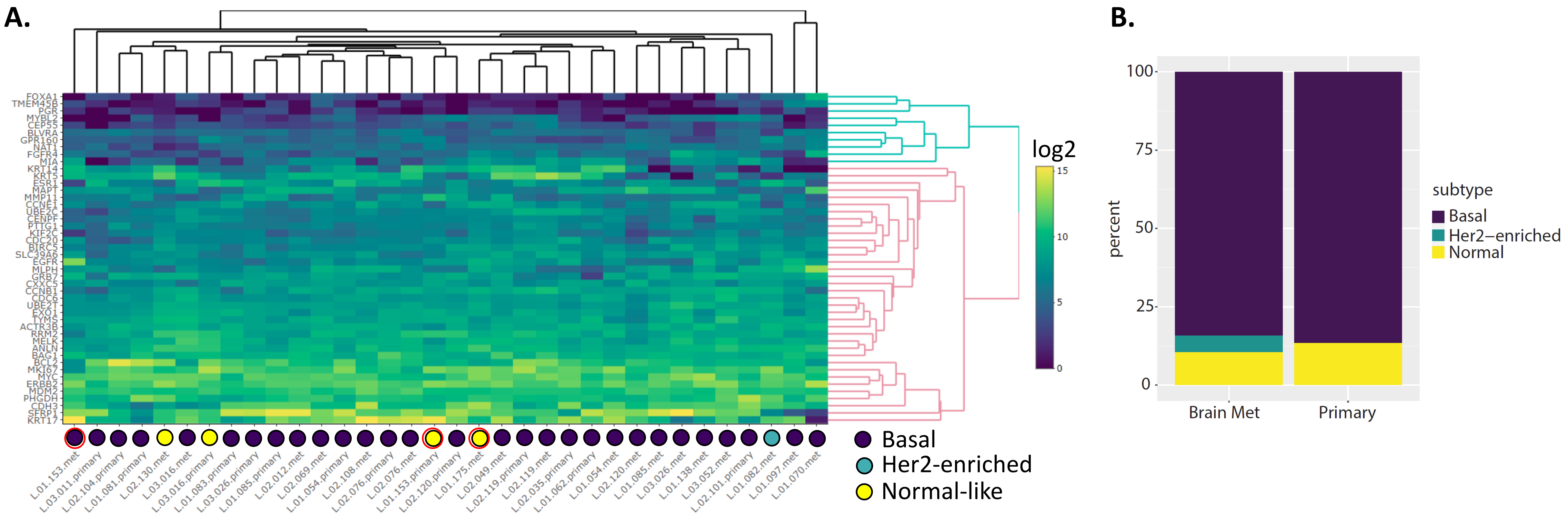

Supplemental Figure 3

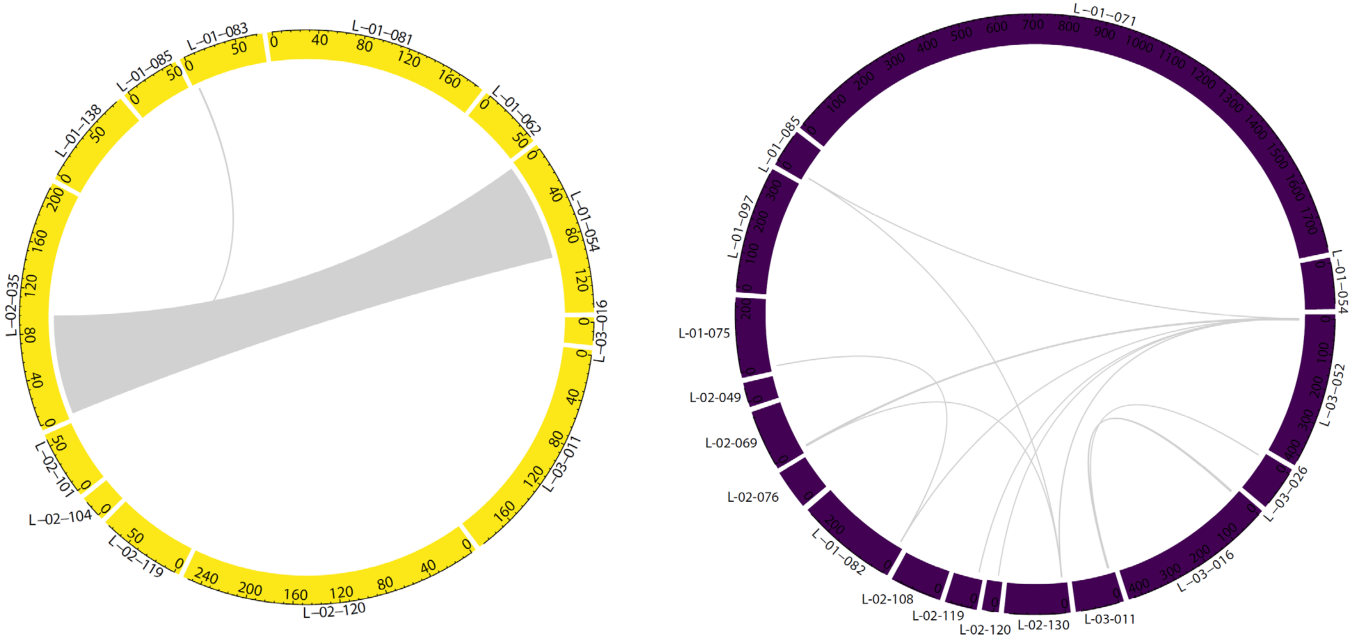

Supplemental Figure 4

Dx primary to death

Variable	OR	Lower.95CI	Upper.95CI	p	Sig.code
TMB	0.3405	0.10551	1.099	0.0715	†
RaceAfrican_American	52.3318	0.55942	4895.477	0.0874	†
StageIII_IV	16.7753	0.06355	4428.323	0.3216	ns
Age	1.6287	1.09044	2.433	0.0172	*

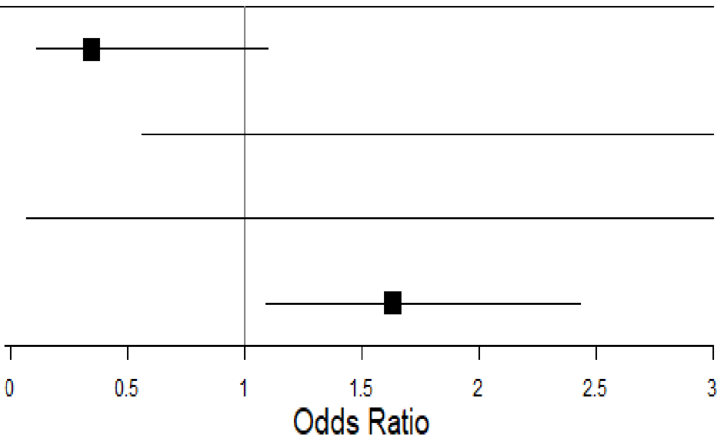

Supplemental Figure 5

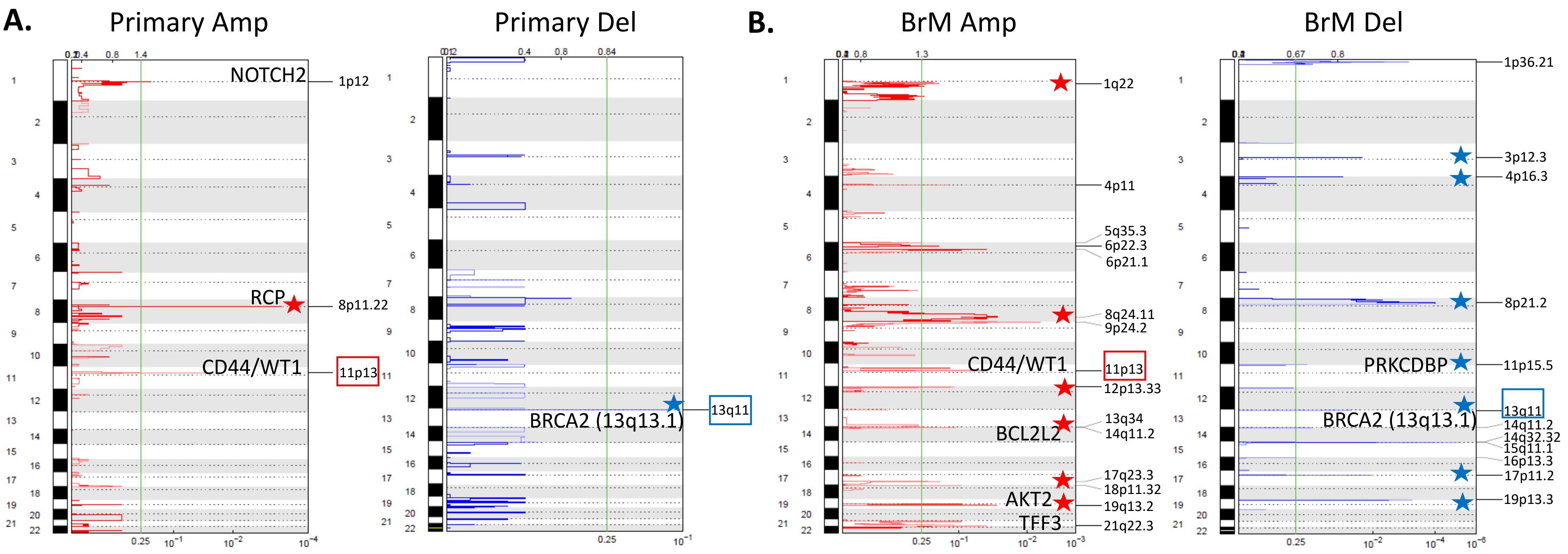

Supplemental Figure 6

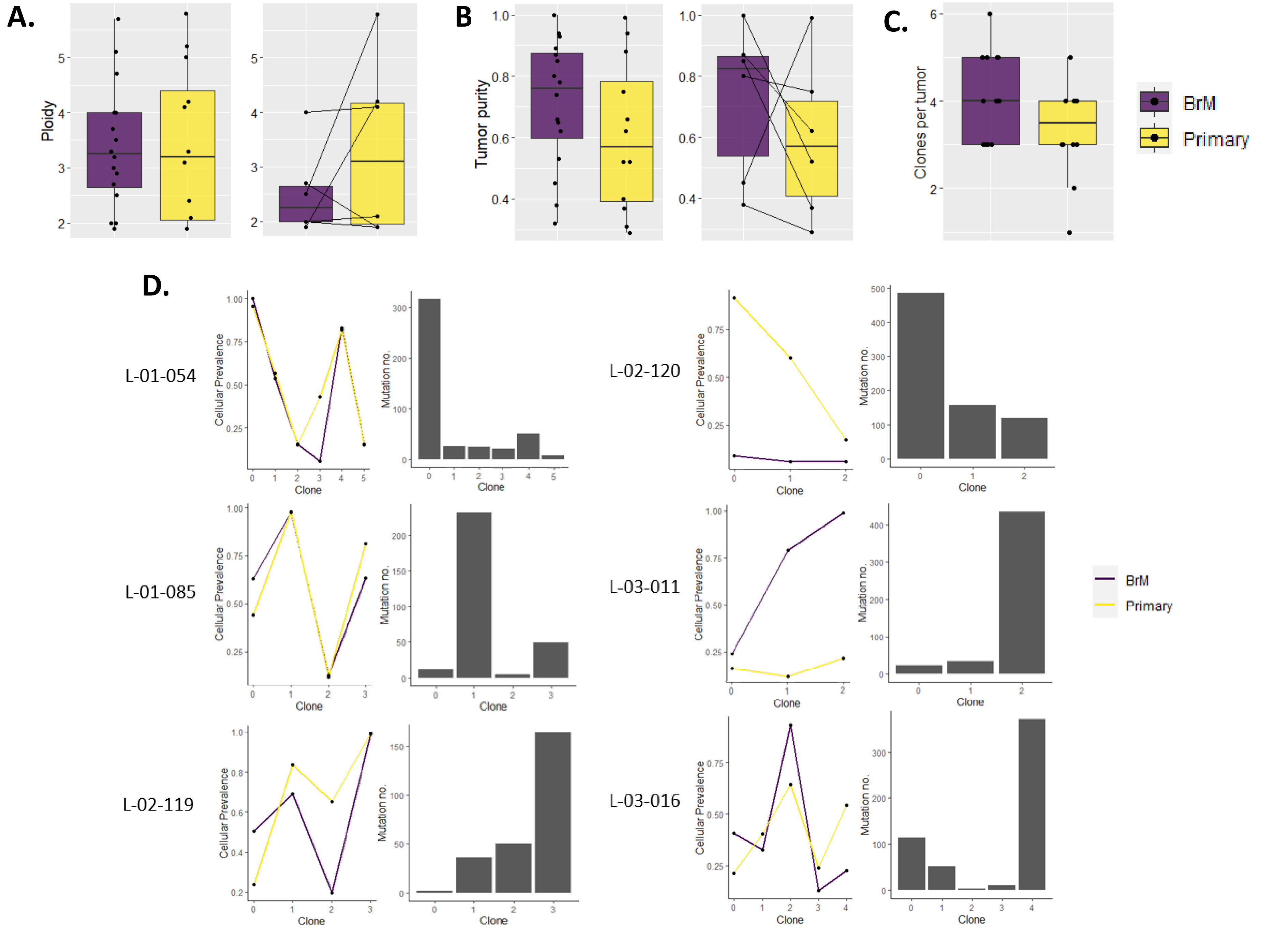

Supplemental Figure 7

A.

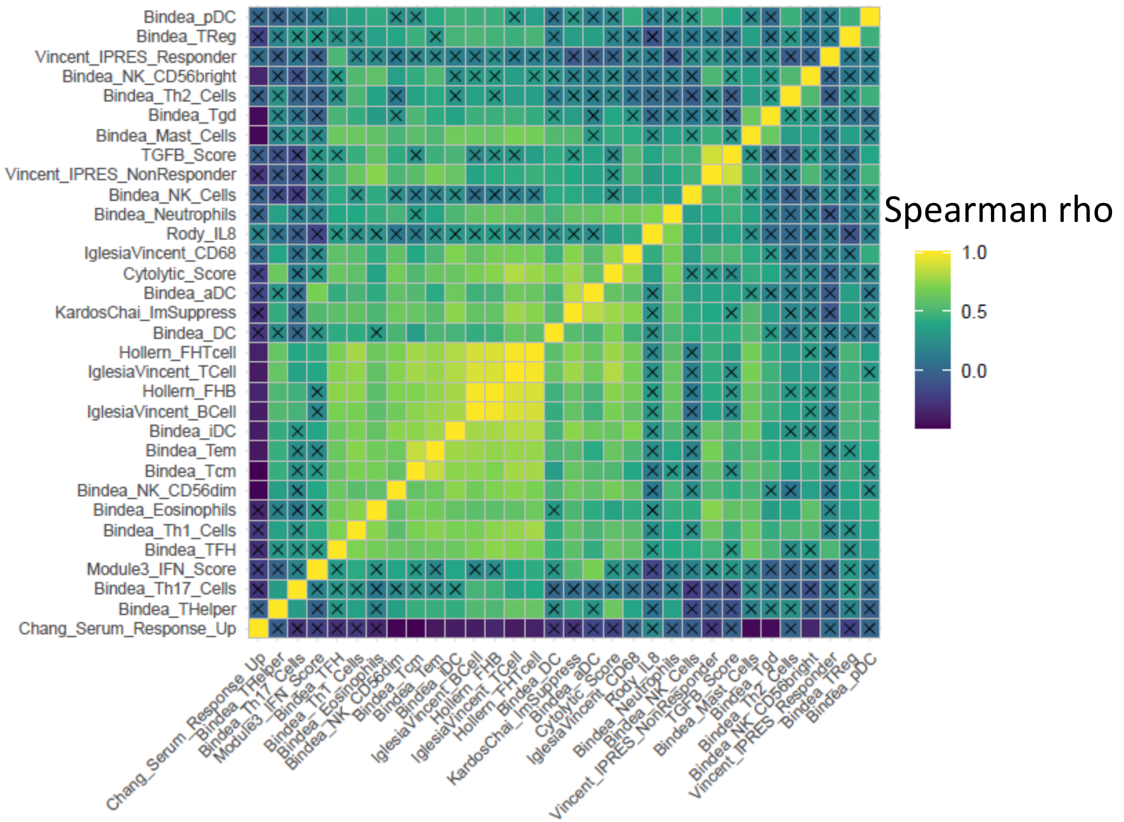

B.

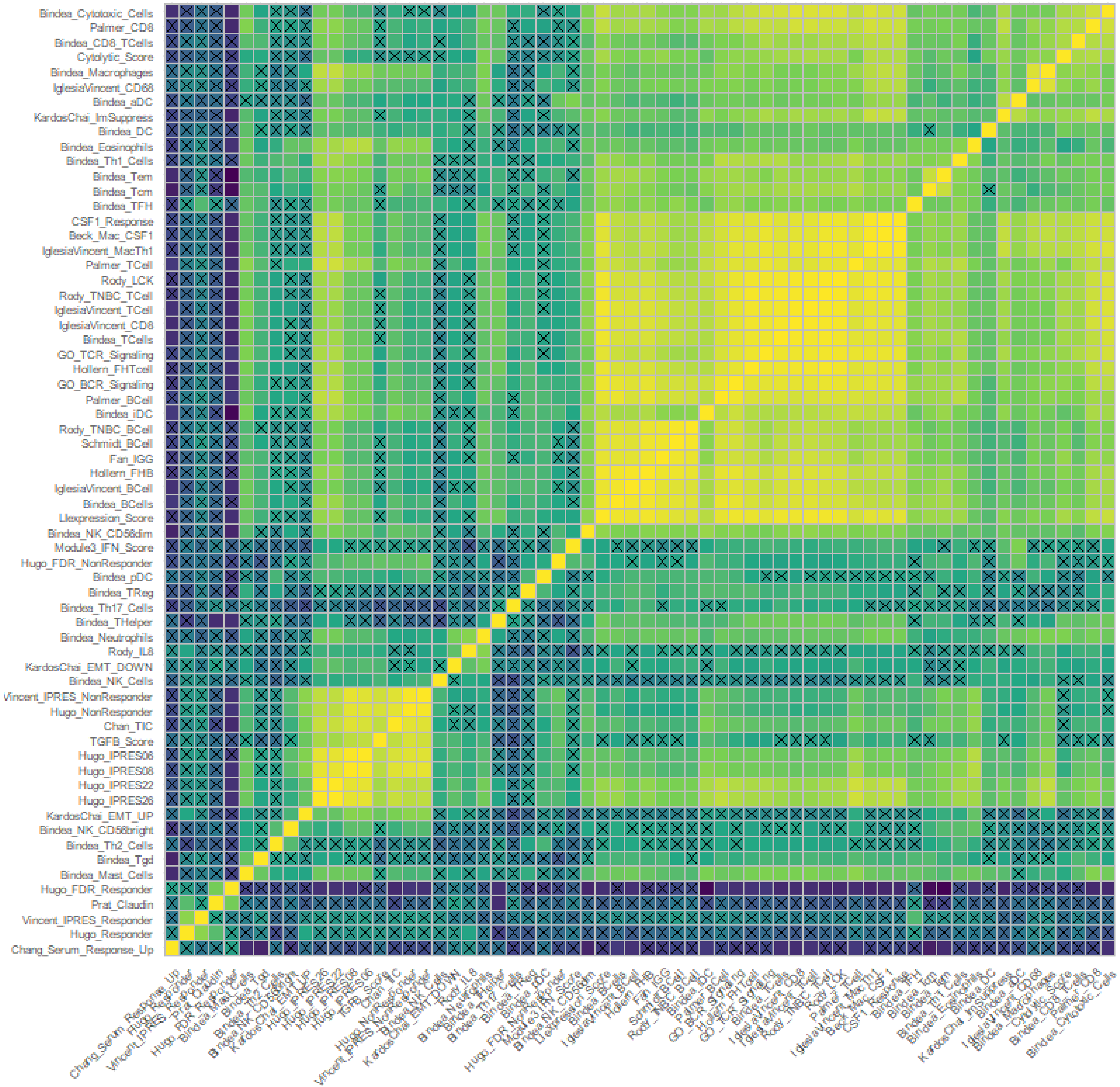

Supplemental Figure 8

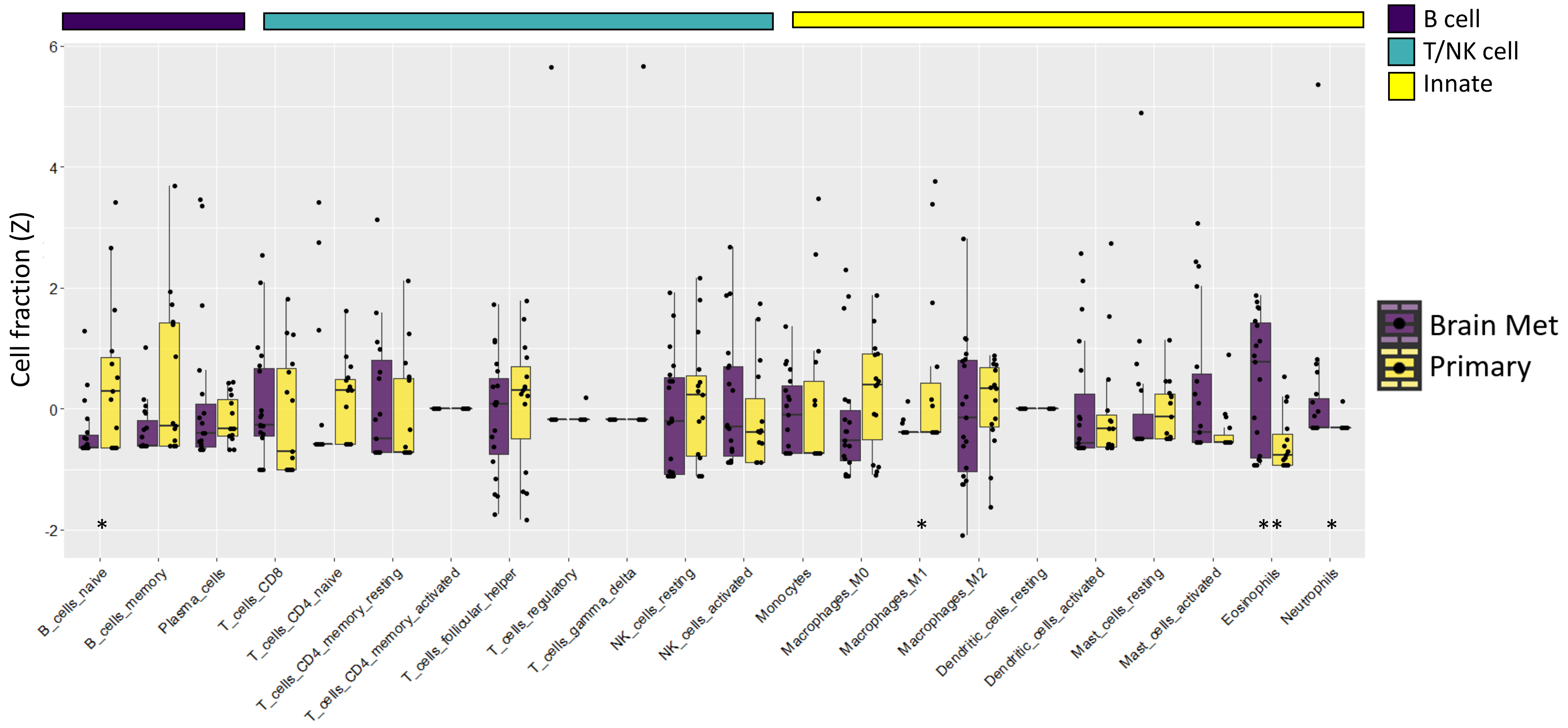

Supplemental Figure 9

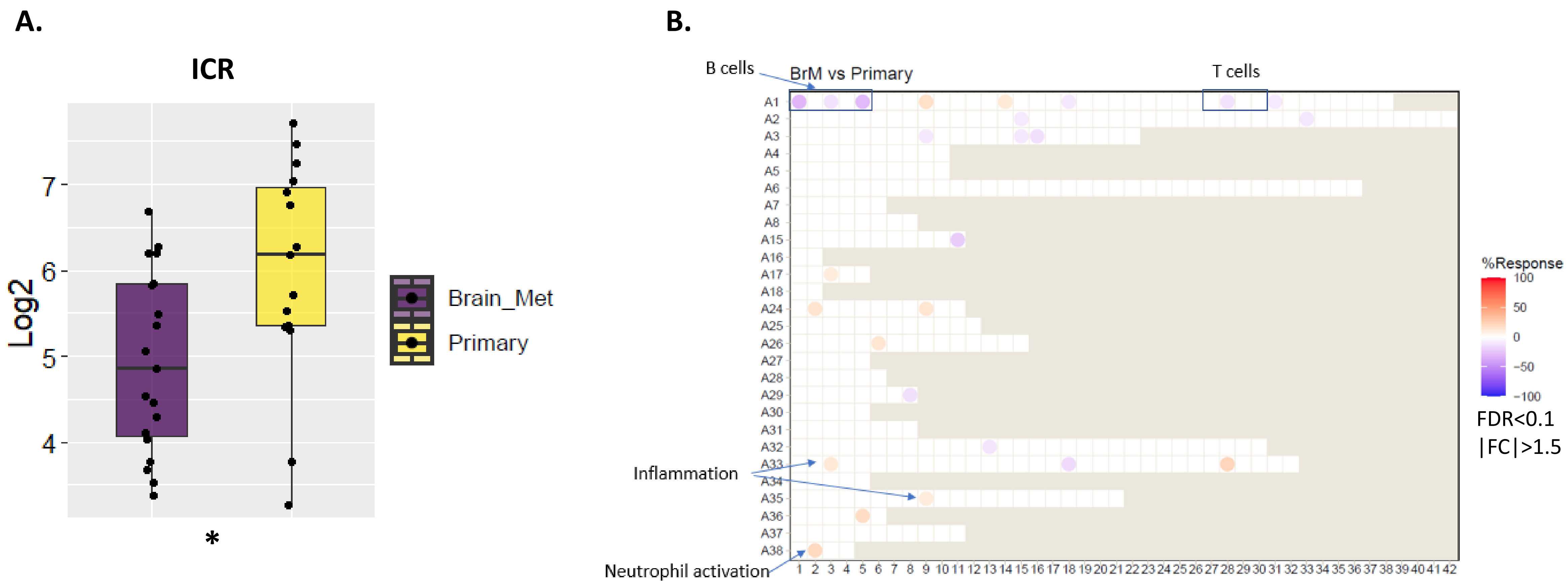

Supplemental Figure 10

Dx primary to Event

Variable	OR	Lower.95CI	Upper.95CI	p	Sig.code
StageIII_IV	1.57472	0.4133	5.999	0.5058	ns
Age	1.06764	1.0028	1.137	0.0405	*
RaceAfrican_American	2.27337	0.6373	8.109	0.2056	ns

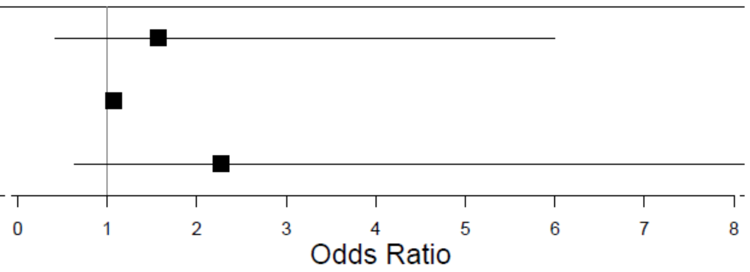

Dx met to Event

Variable	OR	Lower.95CI	Upper.95CI	p	Sig.code
StageIII_IV	0.67513	0.1339	3.405	0.63415	ns
Age	1.10895	1.0294	1.195	0.00648	**
RaceAfrican_American	3.99804	0.7533	21.218	0.10366	ns

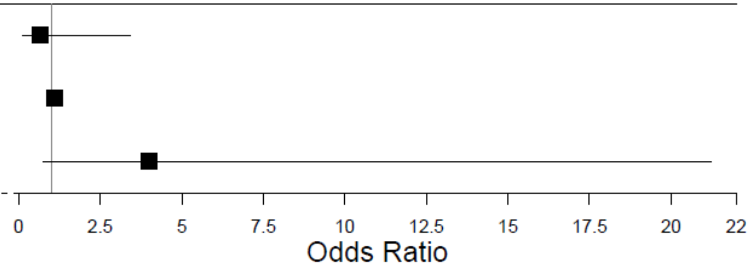

Dx BrM to Event

Variable	OR	Lower.95CI	Upper.95CI	p	Sig.code
StageIII_IV	0.63903	0.1605	2.544	0.5252	ns
Age	1.06816	1.0054	1.135	0.0328	*
RaceAfrican_American	3.24851	0.7755	13.609	0.107	ns

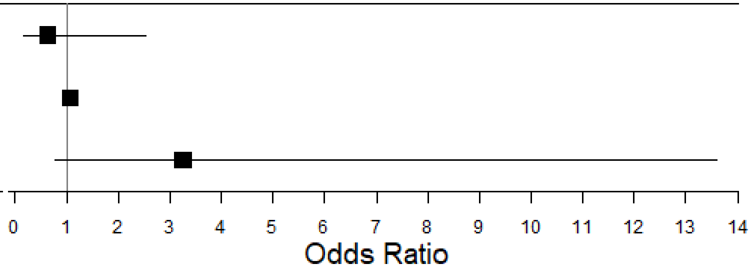

Supplemental Figure 11 **A.**

Dx primary to event/primary tumor IGS

Signature	OR	Lower.95CI	Upper.95CI	p	q
Bindea_Th1_Cells	0.018317504	0.000794796	0.422159955	0.012465906	0.398908992
IglesiaVincent_BCell	0.595483185	0.339350294	1.044938608	0.070800705	0.638819346
Bindea_Mast_Cells	0.160537326	0.020288081	1.270313986	0.083050621	0.638819346

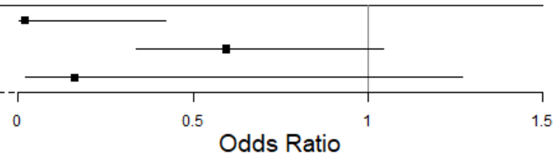

Dx met to event/primary tumor IGS

Signature	OR	Lower.95CI	Upper.95CI	p	q
Bindea_Tem	0.014066371	0.000543787	0.363860557	0.01019645	0.153905938
Bindea_Th1_Cells	0.043732438	0.003265689	0.585642483	0.018072216	0.153905938
Bindea_DC	0.074972374	0.007901063	0.711405192	0.024035135	0.153905938
Hollern_FHTcell	0.212492552	0.05528007	0.816805853	0.024164025	0.153905938
IglesiaVincent_TCell	0.347186474	0.136248856	0.884693284	0.026645202	0.153905938
Cytolytic_Score	0.543649771	0.310628187	0.951475384	0.032830786	0.153905938
IglesiaVincent_BCell	0.372212497	0.149532913	0.926499323	0.033666924	0.153905938
Vincent_IPRES_Responder	0.004012374	1.91E-05	0.843811628	0.043156068	0.172624272
Bindea_THelper	0.042433089	0.001768094	1.018366181	0.051326496	0.182494208
Bindea_iDC	0.098643644	0.008833058	1.101608151	0.059922705	0.191416165
Bindea_Tgd	0.339607463	0.107205423	1.075815255	0.066395612	0.191416165
Hollern_FHB	0.003591698	7.62E-06	1.692849885	0.073076819	0.191416165
Bindea_NK_CD56dim	0.146069927	0.017227241	1.238528199	0.077762817	0.191416165
Bindea_Tcm	0.063296818	0.002512928	1.594349899	0.093621637	0.213992313

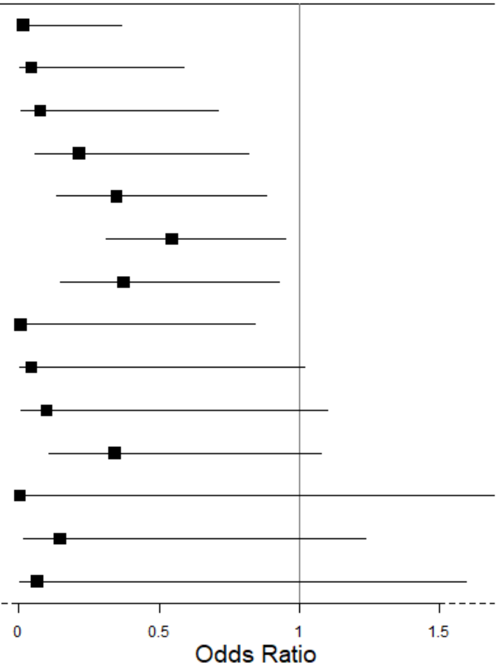

Dx BrMet to event/primary tumor IGS

Signature	OR	Lower.95CI	Upper.95CI	p	q
Bindea_DC	0.062655233	0.007198807	0.54532343	0.012099291	0.387177312
Vincent_IPRES_Responder	0.005426987	3.97E-05	0.741808057	0.037617575	0.487732424
Hollern_FHB	0.654308483	0.421839668	1.01488699	0.058227362	0.487732424
Bindea_Tem	0.154068767	0.018570757	1.278202354	0.083168734	0.487732424
Bindea_Th1_Cells	0.172158222	0.022556238	1.313980334	0.089765614	0.487732424

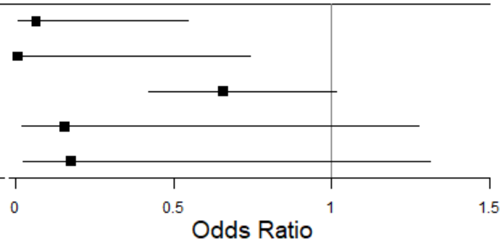

Supplemental Figure 11 (continued)

B.

Dx primary to event/BrMet IGS

Signature	OR	Lower.95CI	Upper.95CI	p	q
Bindea_Th17_Cells	0.248706142	0.085590554	0.722681912	0.010566106	0.195795845
Bindea_Neutrophils	0.445130992	0.23046732	0.859738379	0.015954961	0.195795845
Bindea_Eosinophils	0.080140584	0.009734028	0.659800167	0.018947985	0.195795845
Vincent_IPRES_Responder	0.015256018	0.000332847	0.699258183	0.032091545	0.248709474
Rody_IL8	0.631769913	0.406337708	0.98226971	0.041409186	0.256736953
Bindea_NK_CD56bright	0.217173521	0.046000415	1.025302451	0.05380428	0.27798878
Module3_IFN_Score	1.500364797	0.956499656	2.353471338	0.077339887	0.324349815
Bindea_Tgd	0.606815536	0.338369576	1.088233461	0.093693501	0.324349815
Hollern_FHB	0.76906397	0.565001654	1.046827715	0.095103933	0.324349815

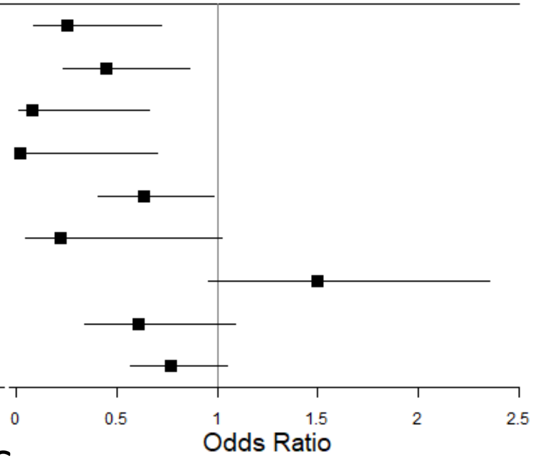

Dx met to event/BrMet IGS

Signature	OR	Lower.95CI	Upper.95CI	p	q
Bindea_Tgd	0.376661451	0.172715623	0.821430314	0.014110531	0.29651761
Chang_Serum_Response_Up	161.6825059	1.878948741	13912.69073	0.025257016	0.29651761
Vincent_IPRES_Responder	0.006431683	6.84E-05	0.604469481	0.029470151	0.29651761
Bindea_Mast_Cells	0.333854348	0.115293954	0.96673522	0.043142461	0.29651761
Bindea_Th17_Cells	0.336365286	0.114328405	0.989619388	0.047825421	0.29651761
Bindea_Neutrophils	0.476685131	0.208096732	1.091937928	0.079776948	0.412180898

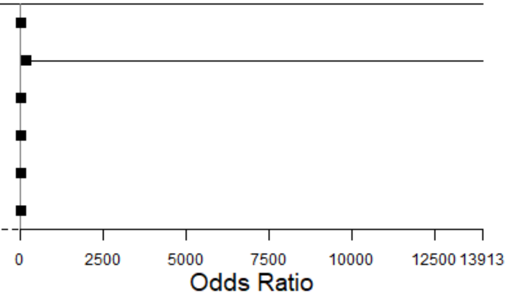

Dx BrMet to event/BrMet IGS

Signature	OR	Lower.95CI	Upper.95CI	p	q
Chang_Serum_Response_Up	680.4145596	6.534931208	70844.50596	0.005924287	0.183652897
Bindea_Tgd	0.382498968	0.177847085	0.822647503	0.013908352	0.215579456
Vincent_IPRES_Responder	0.005943483	5.94E-05	0.594522923	0.029164149	0.301362873
Bindea_Th17_Cells	0.388110038	0.143207039	1.051829593	0.062796932	0.486676223
Bindea_Mast_Cells	0.41608698	0.15077683	1.148242572	0.09044374	0.560751188

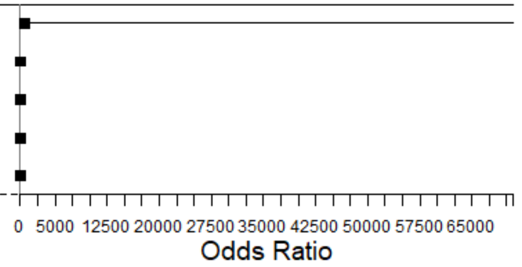

Supplement: Supplemental Figure 1 — Samples included in immunohistochemical and sequencing (RNA and DNA) analyses. Primary breast tumor, BrM, and normal blood specimens from a total of 25 patients with BrM from TNBC were included in the analyses. Matched blood was analyzed by DNA sequencing as a comparison for tumor WES for identification of somatic variants. Final sample numbers by tissue and analysis type are provided. [file DataSheet_1.zip › Supp Figs and Table_1419_for upload.pdf]
